# *CSNK1G2* differently sensitizes tamoxifen-induced decrease in PI3K/AKT/mTOR/S6K and ERK signaling according to the estrogen receptor existence in breast cancer cells

**DOI:** 10.1371/journal.pone.0246264

**Published:** 2021-04-16

**Authors:** Anh Thu Nguyen Hoang, Kwang-Lae Hoe, Sook-Jeong Lee

**Affiliations:** 1 Department of Bioactive Material Sciences, Jeonbuk National University, Jeonju, Jeollabuk-do, Republic of Korea; 2 Department of New Drug Discovery and Development, Chungnam National University, Daejeon, Republic of Korea; University of Hawai’i at Manoa, UNITED STATES

## Abstract

Tamoxifen (TAM) is a selective estrogen receptor modulator used for breast cancer patients. Prolonged use of tamoxifen is not recommended for some patients. In this study, we aimed to identify molecular targets sensitive to TAM using a genome-wide gene deletion library screening of fission yeast heterozygous mutants. From the screening, *casein kinase 1 gamma 2* (*CSNK1G2*), a serine-/threonine protein kinase, was the most sensitive target to TAM with a significant cytotoxicity in estrogen receptor-positive (ER^+^) breast cancer cells but with only a slight toxicity in the case of ER^-^ cells. In addition, tumor sphere formation and expression of breast stem cell marker genes such as *CD44*/*CD2* were greatly inhibited by *CSNK1G2* knockdown in ER^+^ breast cancer cells. Consistently, *CSNK1G2* altered ERα activity via phosphorylation, specifically at serine (Ser)^167^, as well as the regulation of estrogen-responsive element (ERE) of estrogen-responsive genes such as *CTSD* and *GREB1*. However, *ERα* silencing almost completely blocked *CSNK1G2*-induced TAM sensitivity. In ER^+^ breast cancer cells, combined treatment with TAM and *CSNK1G2* knockdown further enhanced the TAM-mediated decrease in phosphatidylinositol 3-kinase (PI3K)/AKT/mammalian target of rapamycin (mTOR)/ribosomal protein S6 kinase (S6K) signaling but not extracellular signal-regulated kinase (ERK) signaling. Inversely, in ER^-^ cells treated with TAM, only ERK and PI3K signaling was altered by *CSNK1G2* knockdown. The CK1 inhibitor, D4476, partly mimicked the *CSNK1G2* knockdown effect in ER^+^ breast cancer cells, but with a broader repression ranging from PI3K/AKT/mTOR/S6K to ERK signaling. Collectively, these results suggest that *CSNK1G2* plays a key role in sensitizing TAM toxicity in ER^+^ and ER^-^ breast cancer cells via differently regulating PI3K/AKT/mTOR/S6K and ERK signaling.

## Introduction

Breast cancer is one of the most common cancers in women [1]. The mortality rate of breast cancer dropped, owing to the established system of cancer screening and early comprehensive diagnosis. Nevertheless, difficulties persist in obtaining the right treatment methods because cancer is highly heterogeneous and is accompanied by diverse clinical features [2]. Breast cancer can be managed in several ways; particularly, hormonal therapy, chemotherapy, and monoclonal antibodies are commonly used. According to molecular heterogeneity profiling, breast cancer cells can be largely characterized into five intrinsic subtypes: 1) luminal type A expressing high estrogen receptor alpha (ERα) together with progesterone receptor (PR); 2) luminal type B expressing low ERα along with high human epidermal growth factor 2 (HER2); 3) HER2-positive (HER^+^) possessing luminal characteristics but lack ERα; 4) basal type, also called triple-negative meaning ER^-^/PR^-^/HER2^-^; and 5) normal-like expressing high levels of alcohol dehydrogenase 1B (ADH1B), similar to luminal type A [2,3]. Of these subtypes, ER^+^ luminal types account for two-thirds of all breast tumor types [4]. Although the basal type of tumor has the worst progress and prognosis after treatment, endocrine therapy specifically targeting ERα is at the forefront of breast tumor treatment. The first clinical trial of endocrine therapy for breast cancer was in the late nineteenth century and was performed by a British scholar, Beatson, who treated a premenopausal advanced breast cancer patient using oophorectomy [5]. Since then, anti-estrogenic agents have been recognized as a reliable and effective treatment to curtail the growth of estrogen-dependent tumors.

Tamoxifen (TAM) is the earliest non-steroidal selective estrogen receptor modulator and has been widely used since the early 1970s [6]. It stabilizes the tumor through consistent use over a period of 5 years, resulting in a breast cancer recovery rate of more than 50% [4,7]. However, TAM also exhibits an estrogenic effect in certain types of cancer patients. TAM has a triphenylethylene backbone structure that can bind to ERα, thus preventing estrogen from binding to ERα [8]. TAM itself has a weak affinity to ER. It is frequently mentioned as a prodrug, and upon metabolism, it is sequentially converted to active metabolites such as *N*-dimethyl-TAM, 4-hydroxy-TAM, and 4-hydroxy-*N*-desmethyl-TAM (endoxifen), which are known to have higher affinities for ERα than the prodrug [9]. The mechanism of action of TAM can be explained in several ways. First, TAM hinders ER-induced increase in cell proliferation and protein synthesis after binding to activation function 2 (AF2) [10]. Second, TAM can stimulate the process of caspase family proteins by releasing cytochrome c from mitochondrial membrane receptors, leading to apoptosis [11]. Although TAM has many benefits, breast cancer patients are prescribed a combination of TAM and other supplements to reduce the side effects of TAM and enhance the effectiveness of the treatment.

Protein kinases play a central role in target identification for drug development because they are closely associated with signal transduction via regulating the activities of various substrates related to majority of cellular processes. Breast cancer patients possess numerous genomic aberrations, many of which converge on several pathways involved in cancer cell signal transduction, such as phosphatidylinositol 3-kinase (PI3K)/AKT/mammalian target of rapamycin (mTOR) and the extracellular signal-regulated kinase (ERK) cascades [12]. There are multiple signal intersections and feedback loop among the molecules, which is suggestive of their functional importance in normal physiology. Therefore, unraveling the complex cross-talk between these signal molecules may play a pivotal role in solving the etiology of various tumors.

Casein kinases (CSNK) 1, a unique group within the superfamily of serine/threonine specific protein kinases, function as regulators of signal transduction pathways in eukaryotic organisms from yeast to humans [13]. CSNK1 can be divided into CSNK1A, CSNK1B, CSNK1D, CSNK1G1, CSNK1G2, CSNK1G3, and CSNK1E subfamilies. Their functions in cell survival and tumorigenesis were recently reported [7,14]. Of CSNK1 subtypes, the functional role of CSNK1A, CSNK1D, and CSNK1E in different cancer types have also been reported [15]. CSNK1A controls the mitotic spindle formation during cell division, thus regulating cell cycle during M phase in mouse oocytes [16]. CSNK1A also regulates mTOR activity by altering the stability of DEPTOR, the endogenous mTOR inhibitor [17]. However, the role of the CSNK1G isoforms, especially, CSNK1G2, has yet to be investigated in depth. Recently, the function of CSNK1G1 and CSNK1G3 in tumor necrosis factor (TNFα)-induced necroptosis, an important necrotic cell death under apoptosis-deficient conditions, has been reported in human colon cancer [7].

Fission yeast *Schizosaccharomyces pombe* (*S*. *pombe*) is an excellent model system to study cell morphogenesis and cell division cycles. Most genome-wide screening for drugs has been applied with a haploid gene deletion library consisting of only nonessential genes [18]. However, screening using a heterozygous gene deletion library are more powerful because they contain all the genes, including essential genes in addition to nonessential genes [19,20]. Here we constructed a genome-wide gene deletion library in fission yeast, allowing us to find sensitive or resistant target genes against TAM by the principle of drug-induced haploinsufficiency [21]. From the systematical screening, we found the fission yeast *cki3* as the most sensitive target for TAM. Therefore, the main purpose of this study was to explore the potential of *CSNK1G2*, the human ortholog of fission yeast *cki3*, as a novel TAM-sensitive target. We report the function of *CSNK1G2* in breast cancer cells in TAM sensitivity, specifically comparing its effects in ER^+^ and in ER^-^ breast cancer cells.

## Materials and methods

### Chemicals and antibodies

4-hydroxy-TAM (#H7904, Lot#086M4013V) and β-estradiol (E_2_; #E2257) were obtained from Sigma-Aldrich (St. Louis, MO, USA). D4476 (#74014) was purchased from Stem Cell Technologies Inc. (Cambridge, MA, USA). Antibodies against progesterone receptor (PR; #8757), HER2 (#2242), phospho-ERα (p-ERα) (Ser^118^, #2511, Ser^167^, #64508;), AKT (#4691), phosphor-AKT (Tyr^308^, #4060), mTOR (#2972), phosphor-mTOR (Ser2448, #5536), PI3 kinase p85α (#13666), phosphor-PI3 kinase p85 (Tyr^458^)/p55 (Tyr^199^) (#4228), S6 ribosomal protein (rpS6) (#2217), phosphor-rpS6 (Ser^235/236^) (#4858), ERK1/2 (#4695), and phosphor-ERK1/2 (#4370) were purchased from Cell Signaling Technology (Danvers, MA, USA). Antibodies against CSNK1G2 (#GTX33123) and ERα (#PA1-309) were obtained from GeneTex Inc. (Irvine, CA, USA) and Thermo Fisher Scientific (Waltham, MA, USA), respectively. Tubulin α antibody (#BS1699) was purchased from BioWorld Technology Inc. (Louis Park, MN, USA).

### Genome-wide screening of heterozygous gene deletion library in fission yeast

For systematic screening of target genes sensitive to TAM, we used a previously constructed heterozygous gene deletion library [20]. Briefly, the library was constructed by homologues replacement of each gene into KanMX marker gene as a selection marker based on the parental strain of a SP286 wild-type (*ade6-M210/ade6-M216*, *leu1-32/leu1-32*, *ura4-D18/ura4-D18h*^*+*^*/h*^*+*^). The library was pooled and aliquoted into 100 μL vials for each screen. Vials were kept frozen at -80°C until use. Notably, each deletion mutant had a pair (up- and down-tag) for unique built-in molecular bar coded for a parallel analysis. Systematic screening of TAM target genes was performed as previously reported [19]. Briefly, a vial of frozen pool was activated in 50 ml of YES media for 24–30 h up to OD_600_ = 2 (approximately 4.4 x 10^7^ cells/ml). These cells were then diluted in 50 ml of YES media to OD_600_ = 0.05 and cultivated up to OD_600_ = 1.6 (approximately 3.5 x 10^7^ cells) with or without TAM (15 μM). This was repeated four times every 5 generations up to 20 generations. An aliquot of 3.5 x 10^7^ cells was harvested every 5 generations. Genomic DNA was prepared using a ZR-Fungal/Bacterial DNA kit (Zymo Research, Irvine, CA, USA). Microarray experiment was performed using a custom-made GeneChip (48 K KRIBB_SP2, Thermo Fisher Scientific) and fluorescence-labeled probe prepared by PCR for the pair of bar codes [20]. Three independent microarray experiments were performed. Repeated targets of every top 10 targets sensitive to TAM were selected as the first-line targets for TAM. Finally, 6 target strains were selected by the order of the sensitivity indicated as the relative growth fitness (RF) < 0.9 (p<0.05).

### Confirmation of primary target genes by spotting and liquid assays

Primary candidates were confirmed one by one based on individual growth analysis using a spotting assay. For the spotting assay, cells in log phase were diluted to OD_600_ = 0.5 in YES media and spotted in 5-fold serial dilutions onto YES agar plates with or without 15 μM TAM. First-line targets for TAM were confirmed and subjected to gene ontology (GO) analysis using GO term finder (http://go.princeton.edu/cgi-bin/GOTermFinder).

### Cell culture

MCF-7 (Michigan cancer foundation-7, #HTB-22), and MDA-MB-231 (#HTB-26) breast cancer cell lines were purchased from American Type Culture Collection (ATCC; Manassas, VA, USA). T-47D (#30133) breast cancer cell line was obtained from Korean Cell Line Bank (KCLB; Seoul, Korea). Cell lines were maintained in RPMI-1640 medium supplemented with 10% fetal bovine serum (FBS), penicillin-streptomycin-amphotericin B (100 U/mL, 100 μg/mL, and 250 ng/mL, respectively), and 2 mM L-glutamine at 37°C in a humidified atmosphere containing 5% CO_2_. For experimentation, cells were grown in phenol red-free RPMI 1640 culture medium supplemented with 5% dextran charcoal-treated FBS (Sigma-Aldrich) and 2 mM L-glutamine. Reagents for cell culture were purchased from Life Technologies (Carlsbad, CA, USA) or Welgene (Daegu, Korea), unless otherwise stated.

### siRNA Transfection

Oligonucleotide Silencer^®^ Select siRNAs for knocking down the human genes were purchased from Ambion (Thermo Fisher Scientific). The following siRNA was used: human *CSNK1G2* (siRNA ID: s3631). As a control siRNA, Silencer^®^ Select Negative Control #1 siRNA (#4390843) was used.

Cells were seeded into multi-well culture plates (2×10^4^ cells/well in a 48-well plate; 3.5×10^5^ cells/well in a 6-well plate) and transfected with individual siRNA using Lipofectamine™ RNAiMAX transfection reagent (Life Technologies) following the manufacturer’s instructions. Twenty-four hours after transfection, silenced cells were used for analysis. The efficiency of transfection by siRNA was confirmed either by western blotting or RT-PCR.

### Co-transfection of plasmid DNA and siRNA

pGFP-C1-ERα (GFP-ERα), a mammalian expression of ERα fused to GFP, was purchased from Addgene (#28230; Watertown, MA, USA). Cultured MDA-MB-231 cells (2.5×10^4^ cells/well in a 48-well plate; 2.5×10^5^ cells/well in a 6-well plate) were transiently co- transfected with GFP-C1 (Dr. Jungeun An, Jeonbuk National University, Jeonju, South Korea), as a control construct, or GFP-ERα, using Lipofectamine™ 3000 (Invitrogen, #L3000008) together with control or *CSNK1G2* siRNA as suggested by the manufacturer. Twenty four hours after transfection, the cells were treated with different combination of drugs.

### Quantitative real-time PCR (qRT-PCR)

Total RNA was extracted from cells using TRIzol Reagent (Life Technologies) according to the manufacturer’s instructions. One microgram of total RNA was reverse transcribed into cDNA using a qPCRBIO cDNA synthesis kit (#PB30.11, PCR Biosystems Inc.; London, UK). qRT-PCR reactions were then performed using 2× qPCRBIO SyGreen Blue Mix Lo-ROX (PCR Biosystems Inc.) as per the manufacturer’s protocol and run on Quantstudio5 Real-time PCR log (Thermo Fisher Scientific; Massachusetts, USA). Specific primer sequences used in this study are listed in S1 Table. Each test was repeated three times, independently.

### Transient transfection and dual-luciferase assay

Luciferase assay for estrogen-responsive element (ERE) activity measurement was performed using a dual-luciferase reporter assay system (Promega, WI, USA) according to the manufacturer’s instructions. In brief, MCF-7 cells were seeded into 96-well plates at a density of 1 × 10^4^ cells/well. Cells were transfected with (ERE)_4_-luciferase plasmid and SV40-*Renilla* luciferase plasmid using Lipofectamine 2000 reagent (Thermo Fisher Scientific). Transfection solutions were replaced after 24 h. Cells were then treated with and without 1 μM TAM and further incubated for 24 h prior to cell lysis and measurement of firefly- and *Renilla*-luciferase luminescence using a Stop-Glo reagent system (Promega; WI, USA). Each value was normalized to the *Renilla* luciferase control. Each data point generated represents the average of triplicate determinations.

### 3-(4,5-dimethylthiazol-2-yl)-2,5-diphenyltetrazolium bromide (MTT) assay

MCF-7, T-47D, and MDA-MB-231 cell lines (2×10^4^ cells/well in 48-well plates) transfected with control siRNA or each target siRNA for 24 h were treated with various drugs at indicated concentrations. Their viability was then measured using the MTT assay. Briefly, 25 μL of MTT stock solution (5 mg/mL in phosphate-buffered saline) was added to each well followed by incubation at 37°C for 1 h to allow cell-mediated reduction of MTT. To detect the amount of reduced MTT, media were aspirated and 250 μL of DMSO was added. The absorbance was measured at 562 nm using a micro-plate reader (Epoch™ Microplate Spectrophotometer, BioTek Instruments; Winooski, VT, USA).

### Western blot analysis

Protein expression levels in whole cell extracts of cultured human cancer cells were measured using western blot analysis. Briefly, cells were washed with cold phosphate-buffered saline (PBS) prior to lysing with RIPA buffer (Thermo Fisher Scientific), containing protease and phosphatase inhibitor cocktail. After centrifugation, the supernatant was collected. Protein concentrations in the supernatant were measured by bicinchoninic acid (BCA) method (Thermo Fisher Scientific). For western blotting, equal amounts of proteins were resolved by sodium dodecyl sulfate-polyacrylamide gel electrophoresis and transferred to polyvinylidene difluoride membranes. These membranes were blocked with Tris buffered saline with Tween 20 containing 5% skim-milk for 1 h at room temperature (RT), and incubated overnight with specific primary antibodies at 4°C. The next day, membranes were incubated with HRP-conjugated second antibodies for 1 h at RT. Finally, immunoreactive proteins were visualized using an enhanced chemiluminescence kit (Thermo Fisher Scientific). Protein expression was quantitatively assessed by densitometric analysis of the intensity of each protein band using an ImageJ software. All experiments were repeated at least three times using cultures from different passages.

### Tumor sphere formation assay

Prior to tumor sphere culture, MCF-7, T-47D, and MDA-MB-231 cells were grown as monolayer according to the supplier’s recommendations on tissue culture-treated 10 cm dishes (STEMCELL technologies Inc., Vancouver, BC, Canada). After reaching confluence, cells were seeded onto a 6-well ultra-low adherent plate (STEMCELL Technologies Inc., Catalog #27145) at a density of 1×10 ^4^ cells/well for individual breast cancer cells. Immediately after seeding, *NC* and *CSNK1G2* siRNAs were transfected as mentioned previously. At 24 h after transfection, cells were treated with the drugs. Size and numbers of tumor spheres were observed daily. The number of tumor spheres over 100 μm in size or larger were used for analysis.

### Statistics

All data are presented as mean (SD). For multiple comparisons among groups, one-way analysis of variance followed by a Fisher LSD post-hoc test was employed. Paired t-tests were used to analyze differences between two groups. SigmaPlot (spw8) and GraphPad Prism5 were used for statistical analysis. *P*-values < 0.05 were considered statistically significant.

## Results

### TAM-sensitive targets and their growth inhibitory effects determined by two-rounds of microarray using *S*. *pombe* heterozygotes deletion mutant gene pools and spotting assay

**Fig 1:**
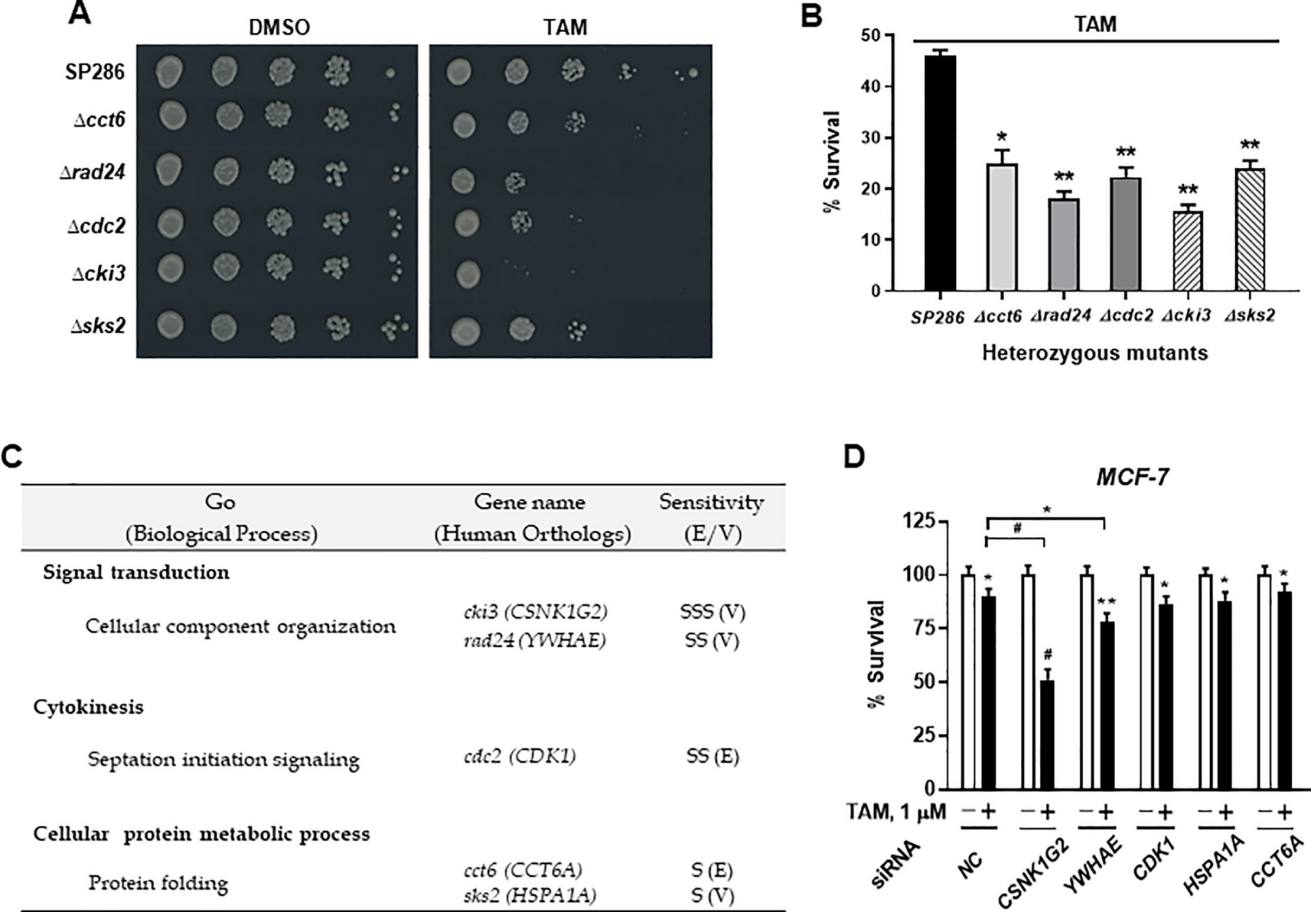
Confirmation of tamoxifen (TAM)-sensitive targets in *Schizosaccharomyces pombe* deletion mutants and cultured human breast cancer cells. (A) TAM sensitivity test using spotting assay for five mutant strains identified in the screening using 15 μM of TAM. Cells in log phase were diluted to OD_600_ = 0.5 in YES media and spotted in 5-fold serial dilutions onto YES agar plates with or without 15 uM of TAM. (B) Quantitative analysis of growth of six different mutants. Relative growths of deletion strains treated with 0 or 15 μM TAM for 9 h were measured as OD_600_ using a microplate reader (mean (SD); n = 5, *P < 0.05, **P < 0.01 *vs*. TAM-treated each control). (C) List of the top 5 TAM targets screened in yeast. Gene ontology (GO) in terms of the biological process was analyzed using GO term finder (http://go.Princeton.edu/cgi-bin/GOTermFinder). Human orthologs were from the HomoloGene or Ensembl database (Http://www.ncbi.nlm.nih.gov/homologene or http://www.ensemble.org). Sensitivity was classified as follows: Severe (SSS), moderate (SS), and mild (S). (D) Bars indicate TAM-induced relative survival rates in candidate target genes-silenced breast cancer cells. MCF-7 cells were silenced with negative control or five individual target siRNAs (*CNSK1G1*, *YWHAE*, *CDK1*, *HSPA1A*, and *CCT6A*) treated with 1 μM tamoxifen for 24 h. Drug-mediated cytotoxicity was measured by MTT assay (mean (SD); n = 4, *P < 0.05, **P < 0.01, ^#^*P* < 0.001 *vs*. each represented counterpart).

Through repeated genome-wide screening against TAM, 10 target strains with RF< 0.9 (*P*<0.05) compared with the wild-type control were selected. Finally, five target mutant strains were confirmed by spotting and liquid assay as shown in Fig 1A and 1B. They consisted of one severe (SSS), two moderate (SS), two mild (S) targets as measured by growth rate against tamoxifen-sensitive toxicity. There were three non-essential targets and two essential targets as measured by gene dispensability. The fission yeast mutant that was most sensitive to TAM was *cki3* deletion mutant (*Δcki3*). According to gene ontology (GO) enrichment analysis for biological process, five target genes were associated with the following processes: signal transduction specifically involved in cellular component organization (*cki3*, *rad24*), cytokinesis like septation initiation signaling (*cdc2*), and cellular protein metabolic process associated with protein folding (*cct6*, *sks2*) (Fig 1C). These results suggest that the cytotoxic effect of TAM might be potentially attributed to a process that results in the biosynthesis of constituent macromolecules, assembly, arrangement of constituent parts, and/or disassembly of a cellular component in fission yeast *S*. *pombe*.

Consistently, when examined whether targets that had been identified by the fission yeast library could represent their effects in human breast cancer cells, ER^+^ MCF-7 cells transfected with siRNAs for five individual target genes (*CSNK1G2*, *YWHAE*, *CDK1*, *HSPA1A*, and *CCT6A*) and tested TAM-sensitive response. Of five candidate target genes, the knockdown of *CSNK1G2* showed the most sensitive tamoxifen toxicity (Fig 1D).

### *CSNK1G2* knockdown differently sensitizes TAM-induced cytotoxicity in ER^+^ and ER^-^ breast cancer cells

**Fig 2:**
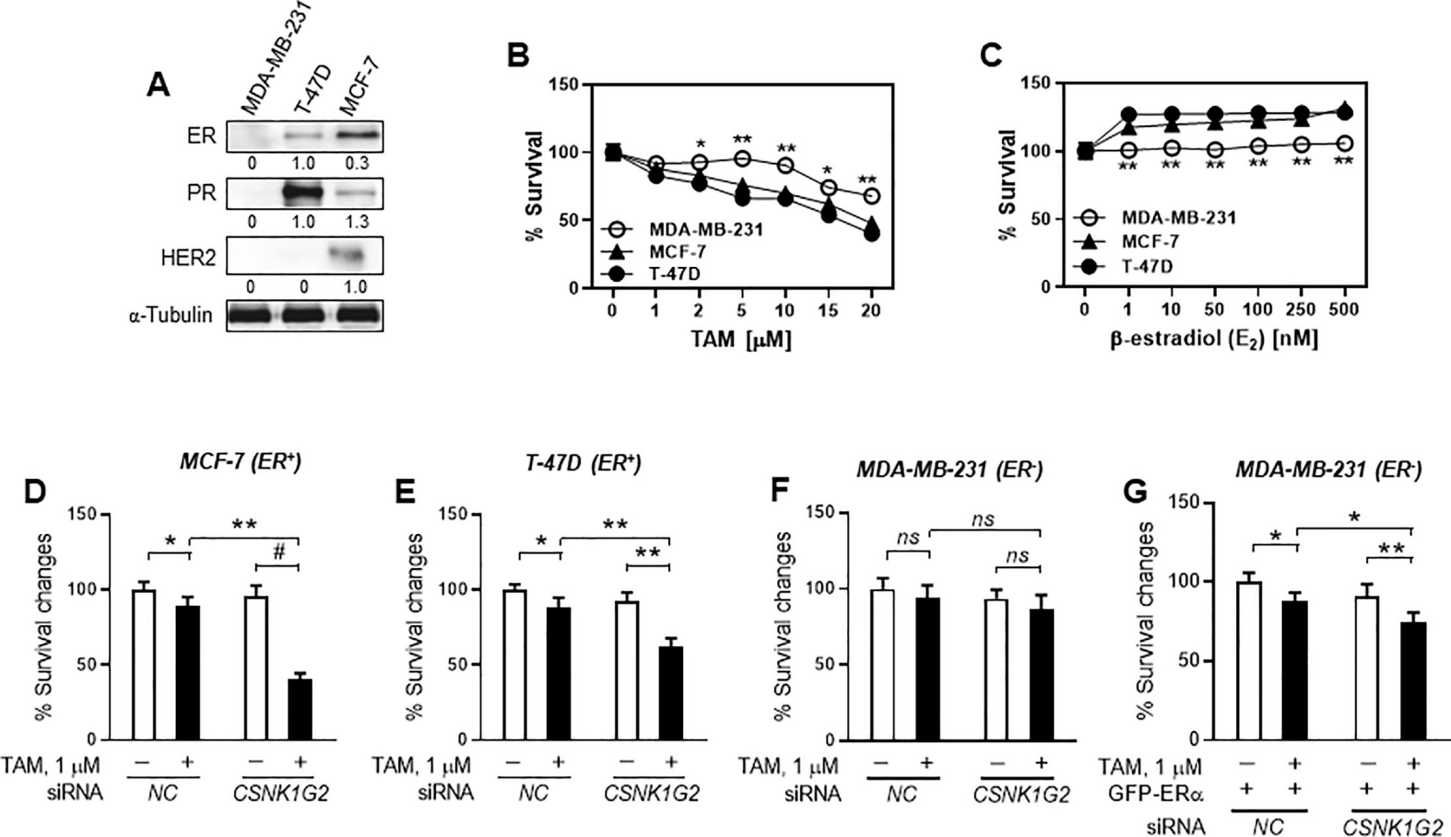
Different TAM sensitivity by *CSNK1G2* knockdown in estrogen receptor (ER)^+^ and ER^-^ human breast cancer cells. (A) Immunoblots for ER, PR, and HER2 in different human breast cancer cell lines. ER^+^ (MCF-7 and T-47D) and ER^-^ (MDA-MB-231) breast cancer cell lines were prepared for western blotting. The expression pattern of different hormone receptors was determined. Protein expression levels were compared to expression levels of α-tubulin; each value under the blots indicates relative protein expression levels determined by densitometric analysis, unless specifically mentioned (mean (SD); n = 4). Dots denote ER^+^ (MCF-7 and T-47D) or ER^-^ (MDA-MB-231) breast cancer cells treated with different concentrations of (B) TAM and (C) β-estradiol (E_2_). Bars denote (D) ER^+^ MCF-7, (E) ER^+^ T-47D, and (F) ER^-^ MDA-MB-231 breast cancer cells treated with TAM after silencing with *NC* or *CNSK1G2* siRNA. (G) ER^-^ MDA-MB-231 also transiently transfected with GFP-C1 or GFP-ERα plasmid DNA as well as with *NC* or *CSNK1G1* siRNA. Twenty-four hours after transfection, cells were treated with vehicle or 1 μM TAM for 24 h. Cellular toxicity was then measured using the MTT assay (mean (SD); n = 4; *P < 0.05, **P < 0.01, ^#^*P* < 0.001 *vs*. each represented counterpart).

The goal of this study was to find an effective target to TAM-treated human breast cancer cells. Prior to conducting the study, we first identified the types of hormone receptors expressed by each breast cancer cell line. Western blot analysis showed that MCF-7 cells were triple positive (ER^+^/PR^+^/HER2^+^), but T-47D cells expressed only ER and PR, and MDA-MB-231 cells were triple negative (ER^-^/PR^-^/HER2^-^). Cytotoxicity assay following treatment with various concentrations of the ER agonist, E_2_, or the selective estrogen receptor modulator (SERM), TAM, in three different cell lines revealed that ER^-^ MDA-MB-231 cells were the least sensitive to both drugs (Fig 2B and 2C; open circles); however, ER^+^ MCF-7 and T-47D cells showed similar viability to both drugs (Fig 2B and 2C).

Since we examined the presence of hormone receptor and responsiveness of the cells to the SERM TAM, we further investigated whether these cell lines present different TAM sensitivity following *CSNK1G2* knockdown. For this, transfected cells with *NC* or *CSNK1G2* siRNA were treated with 1 μM TAM for 24 h and cell viability tests were performed. The efficiency of the gene knockdown in the breast cancer cells was confirmed by western blot analysis (S1 Fig). Interestingly, *CSNK1G2* silencing significantly enhanced TAM-induced cellular toxicity in ER^+^ cells, but not in ER^-^ cells (Fig 2D–2F). However, overexpression of ERα in ER^-^ MDA-MB-231 cells using GFP-ERα plasmid DNA completely reproduced the *CSNK1G2* silencing-induced cytotoxic effects of ER^+^ breast cancer cells (Fig 2G).

### *CSNK1G2* silencing accelerates the suppressive effect of TAM in tumor sphere formation and expression of breast stem cell marker genes in ER^+^ breast cancer cells

**Fig 3:**
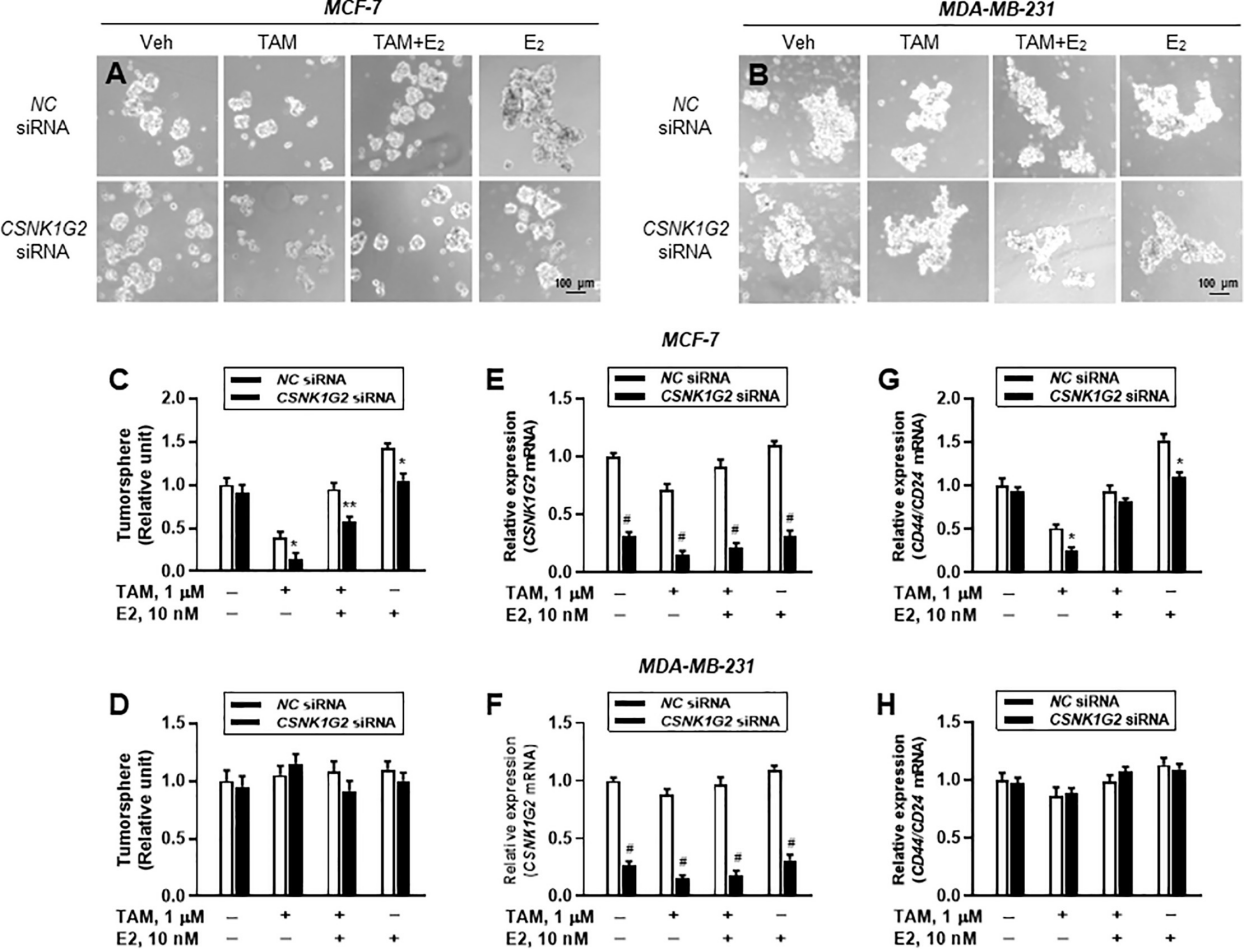
Suppressed tumor sphere formation and expression of cancer stem cells by *CSNK1G2* knockdown in ER^+^ breast cancer cells. Representative images of tumor spheres in *NC* or *CSNK1G2* siRNA-transfected (A) MCF-7 and (B) MDA-MB-231 after treatment with vehicle, 1 μM TAM alone, 1 μM TAM plus 10 nM E_2_, or 10 nM E_2_ alone. Both cells were plated in ultralow-attachment 6-well plates at a density of 2,000 cells/well and cultured in tumor sphere medium with or without each drug for 7 days 24 h after transfection. Quantitative analysis of tumor sphere numbers in (C) MCF-7 and **(**D) MDA-MB-231 cells. Tumor size and numbers were analyzed by ImageJ software. (E, F) *CSNK1G2* knockdown efficiency and (G, H) the ratio of *CD44*/*CD24* mRNA in MCF-7 and MDA-MB-231 was quantified by RT-qPCR. Data are presented as means (SD) (n = 4); *P < 0.05, **P < 0.01, ^#^*P* < 0.001 *vs*. each represented counterpart.

Cancer cells have stem cell-like characteristics, which may play a critical role in the heterogeneity of tumor cells in response to anti-cancer agents and drug resistance [22]. In the present study, we examined whether *CSNK1G2* was associated with altered sphere-forming ability by TAM in human breast tumor cells. ER^+^ (MCF-7) and ER^-^ (MDA-MB-231) human breast cancer cells seeded onto a 6-well ultra-low adherent plate were treated with 1 μM TAM after silencing *CSNK1G2*, and gene-targeting effect was quantified by real-time PCR for *CSNK1G2* (Fig 3E and 3F). For the quantitative analysis, tumor spheres over 100 μm in diameter were considered real tumor spheres with tumor-like characteristics. Consistent with the previous cytotoxicity assay results (Fig 2), the two cell lines showed different responsiveness. TAM (1 μM) significantly reduced the number of formed tumor spheres by approximately 40% in ER^+^ breast cancer cells, whereas *CSNK1G2* silencing itself did not alter the formation of ER^+^-MCF-7 tumor spheres (Fig 3A–3C). When MCF-7 cells were co-treated with 1 μM TAM and *CSNK1G2* silencing, the number of formed tumor spheres was markedly reduced to 15%, compared to that of vehicle-treated cells (Fig 3A–3C). In ER^—^MDA-MB-231 cells, siRNA-mediated gene targeting did not significantly affect TAM-induced decrease in the formation of tumor spheres (Fig 3B–3D).

In the same line, determination of *CSNK1G2* effect on *CD44*/*CD24* mRNA expression, which are known cancer stem cell markers, showed that *CSNK1G2* silencing in MCF-7 cells significantly accelerated TAM-mediated decrease in the ratio of *CD44*/*CD24* mRNA expression to a greater extent than in *NC* siRNA-transfected cells (Fig 3G). However, this *CSNK1G2* silencing-induced effect was not shown in ER^-^ breast cancer cells (Fig 3H). Since the role of *CD44* and *CD24* in tumor metastasis and invasiveness has previously been reported [23], *CSNK1G2* may, in part, contribute to tumor progress.

### TAM-mediated ERE or ER-responsive gene transcriptional activity as well as ERα activation are altered by *CSNK1G2* silencing in ER^+^ breast cancer cells

**Fig 4:**
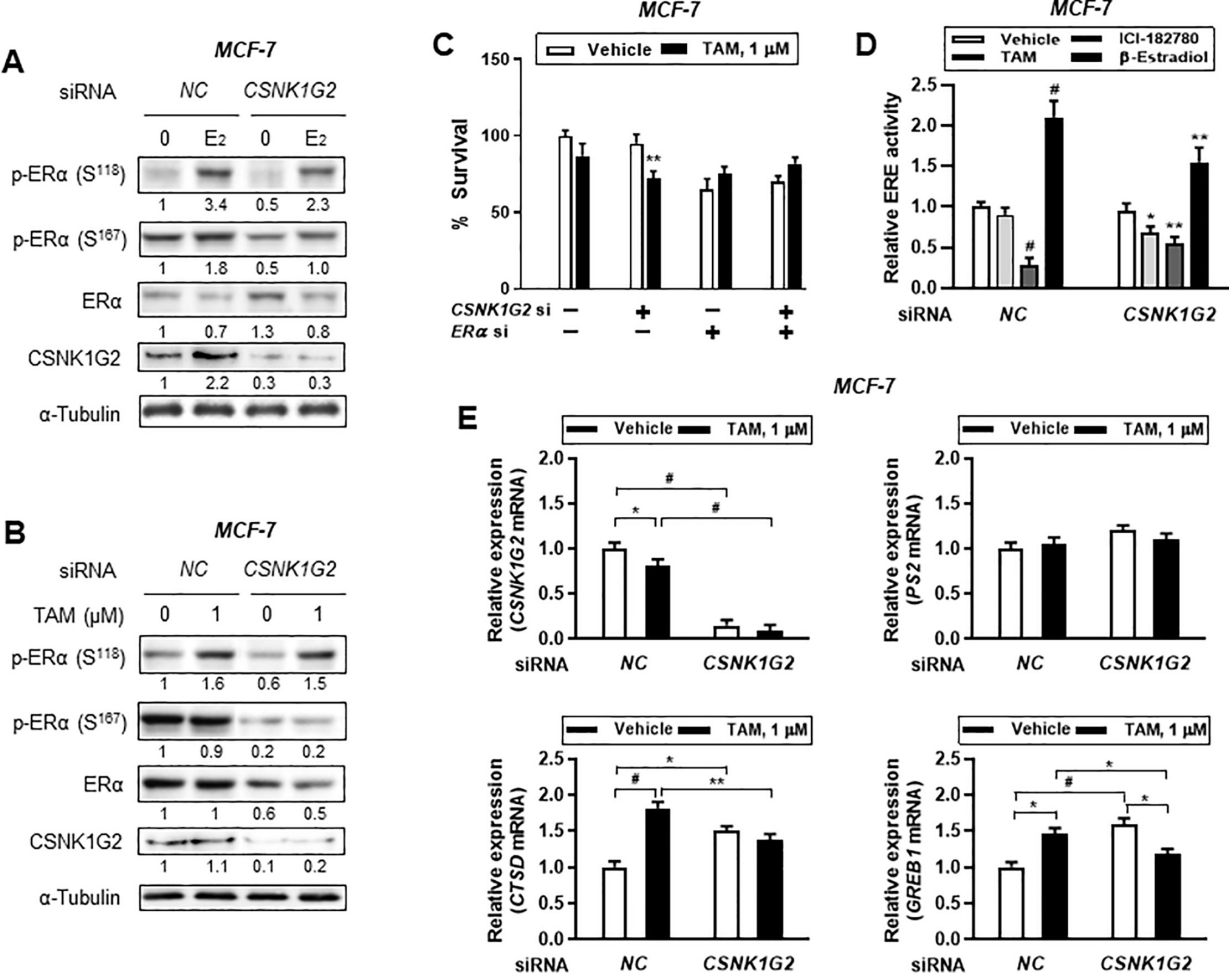
Effect of *CSNK1G2* on TAM-induced ER activity in ER^+^ breast cancer cells. Immunoblots for phosphor-ER (p-ER) in *CSNK1G2* siRNA-transfected breast cancer cells after treatment with (A) 10 nM E_2_ or (B) 1 μM TAM for 24 h. Protein expression levels based on individual bands of phosphor form were compared to expression levels of α-tubulin and/or pan (total) protein. *CSNK1G2* silencing efficiency was proved by western blotting for CSNK1G2. Each value under the blots indicates relative protein expression levels determined by densitometric analysis. (C) Cytotoxicity assay of *CNSK1G2*-silenced ER^+^ breast cancer cells. Cells transfected with different combinations of siRNA, such as *NC* siRNA alone, *CSNK1G2* siRNA alone, *ERα* siRNA alone, or *ERα* plus *CSNK1G2* siRNA, were treated with 1 μM TAM for 24 h; cellular toxicity was then measured using the MTT assay. (D) ERE-luciferase reporter assay showing a significant change in TAM-treated MCF-7 cells. *CSNK1G2* knockdown by siRNA transfection in MCF-7 cells slightly reduced 10 nM estradiol, 1 μM ICI-182780, or 1 μM TAM-mediated ERα transcriptional activity. (E) Quantitative analysis for mRNA expression in MCF-7 cells. Knock-down cells with *NC* or *CSNK1G2* siRNA were treated with vehicle or 1 μM of TAM for 24 h. Transcriptional patterns of diverse ER-responsive genes, *PS2*, *CTSD*, and *GREB1*, were analyzed by RT-qPCR in *CSNK1G2*-silenced ER^+^ breast cancer cells and were compared with those in *NC* siRNA-transfected control. All values were normalized to those in the *NC* siRNA-transfected vehicle group. *GAPDH* was used as a loading control and *CSNK1G2* knockdown efficiency was also confirmed. Data are presented as mean (SD); n = 4; *P < 0.05, **P < 0.01, ^#^*P* < 0.001 *vs*. each represented counterpart.

Estrogen-ERα binding is very important for genomic and non-genomic activation of cellular signaling molecules; thus, the regulation of nuclear gene expression was induced to proliferate or differentiate the breast cancer cells. Here we examined how *CSNK1G2* knockdown influences E_2_ or TAM-mediated phosphorylation of ERα. The phosphorylation level at different sites of ERα, specifically at serine (Ser)^118^ and Ser^167^, was determined by western blot analysis. *CSNK1G2* silencing significantly reduced E_2_-induced ERα phosphorylation at both sites (Fig 4A) and reduced TAM-mediated ERα phosphorylation only at Ser^167^ (Fig 4B). Hence, ERα activity was influenced by *CSNK1G2*, inversely proving that *ERα* knockdown in ER^+^ cells eliminated *CSNK1G2* effect on TAM-induced toxicity. *CSNK1G2*-mediated alteration of TAM toxicity was completely obstructed by ERα exclusion, implying that *CSNK1G2* and ERα interact with a currently unknown mechanism (Fig 4C).

TAM can compete with estrogens for binding to ERα and can interfere with the recruitment of coactivators, required for ERα-mediated gene expression [24]. To elucidate if the target effects on tumor cell proliferation are associated with ERE activity, we assessed ERE transcriptional activity in MCF-7 cells after treatment with 10 nM E_2_, 1 μM ICI-182780, and 1 μM TAM. E_2_ and ICI-182780 were used as positive and negative regulators of ERE transcriptional activity, respectively. As expected, knockdown of MCF-7 cells with *CSNK1G2* significantly altered E_2_, ICI-182780, or TAM-induced ERE transcriptional activity compared to each *NC* siRNA transfected counterpart group (Fig 4D). These results indicated that increased TAM-sensitivity might occur following *CSNK1G2* knockdown, in part, through alteration of genomic ER effect, associated with ERE site of diverse genes in ER^+^ breast cancer cells. Real-time PCR for ER responsive genes such as *CTSD* and *GREB1* in ER^+^ cells confirmed the role of *CNSK1G2*. Low concentration of TAM-induced increase in *CTSD* and *GREB1* expression, except *PS2*, was almost completely removed by *CSNK1G2* siRNA transfection (Fig 4E).

### *CSNK1G2* contributes differently to TAM-induced PI3K/AKT/mTOR/S6K and ERK signaling in ER^+^ and ER^-^ breast cancer cells

Most cancer cells grow rapidly and proliferate abnormally because of altered cellular signal cascades. Presently, we focused on the phosphoinositide 3-kinase/protein kinase B (AKT)/mammalian target of rapamycin/ribosomal S6 kinase (PI3K/AKT/mTOR/S6K) as well as ERK signaling pathways. These pathways are crucial signal transduction networks in the promotion of tumor initiation and progression.

**Fig 5:**
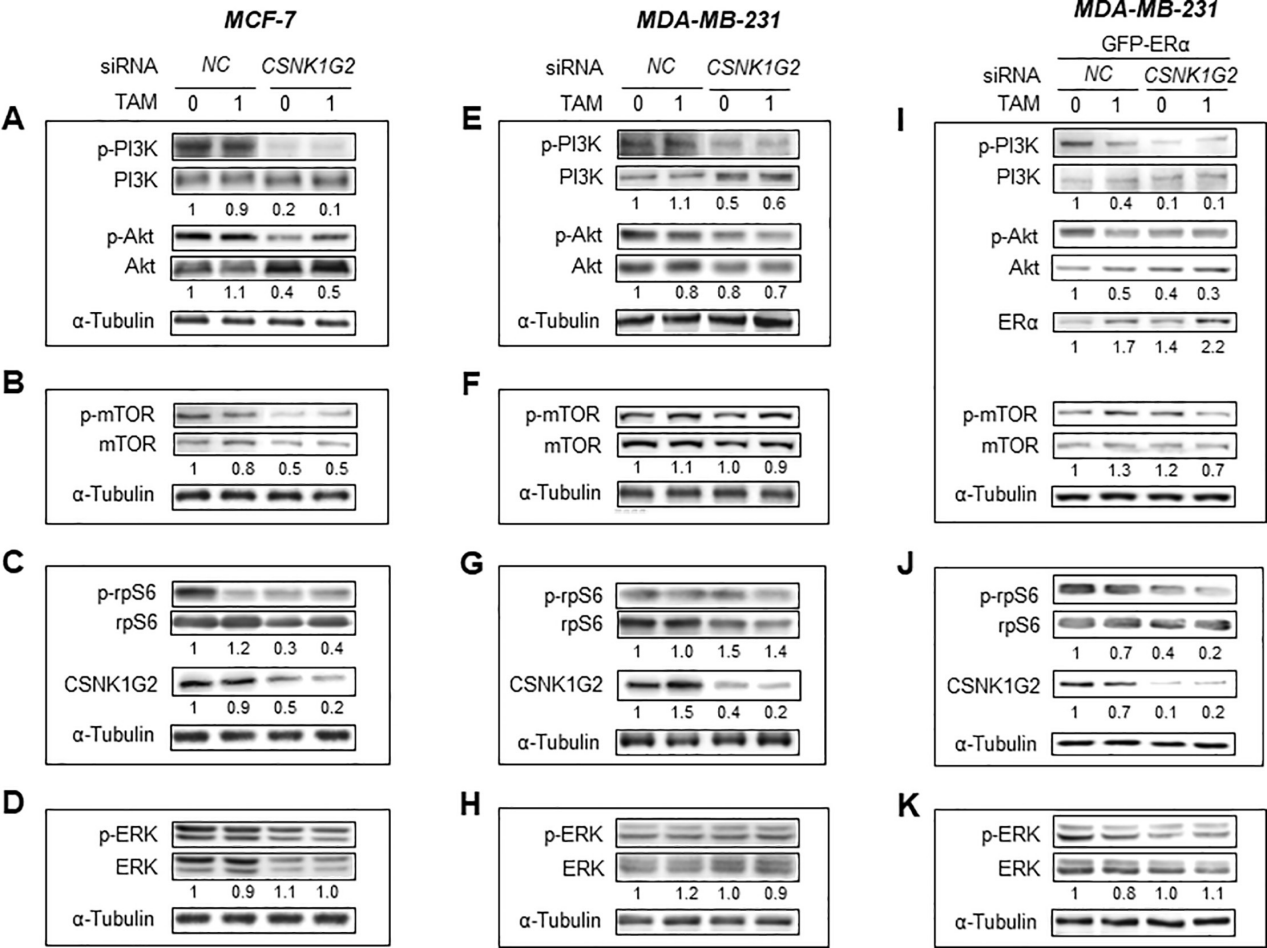
*CSNK1G2* effect on PI3K/AKT/mTOR/S6K and ERK signaling in human breast cancer cells. Immunoblots for PI3K/AKT/mTOR/S6K and ERK signaling-associated proteins in (A-D) MCF-7, **(**E-H) MDA-MB-231, and (I-K) GFP-ERα-overexpressed MDA-MB-231 cells. Western blotting analysis from the breast cells transfected with control siRNA (*NC*) or *CSNK1G2* siRNA (*CSNK1G2*) were performed after treatment with vehicle or 1 μM TAM for 24 h. Protein expression levels based on individual bands of phosphor were compared to expression levels of α-tubulin and/or pan (total) protein. Each value under the blots indicates relative protein expression levels determined by densitometric analysis. Data are presented as mean (SD); n = 4.

Before we examined the *CSNK1G2* silencing-induced signal changes in TAM treated cells, we primarily checked the effect of E_2_ in these pathways (S2 Fig). Interestingly, E_2_ treatment following *CSNK1G2* silencing showed a different effect on AKT/mTOR/S6K signals between ER^+^ and ER^-^ cells, indicating that *CSNK1G2* knockdown significantly inhibited these signals. Consistent with this result, 1 μM TAM in MCF-7 cells slightly reduced the phosphorylation of PI3K but not significantly. However, *CSNK1G2* knockdown significantly inhibited PI3K activity (Fig 5A); these results were also reproduced in the ER^-^ breast cancer cells with less significant reduction (Fig 5E). Simultaneously, activity of AKT, a downstream signal of PI3K, was markedly reduced by both 1 μM TAM and target modulation (Fig 5A). However, in ER^-^ MDA-MB-231 cells, target modulation did not alter AKT activity (Fig 5E). As mTOR is a downstream effector in the PI3K and AKT signaling pathways, we determined whether mTOR activity was altered by modulation of *CSNK1G2* in breast cancer cells. Western blots in MCF-7 cells revealed that *CSNK1G2* silencing reduced ribosomal protein S6 (rpS6), the downstream signaling protein of mTOR, as well as mTOR activity (Fig 5B and 5C). Contrarily, MDA-MB-231 cells transfected with *CSNK1G2* siRNA were not significantly changed in phosphor mTOR (Fig 5F) but rather increased in phosphor rpS6 (Fig 5G). In addition, *CSNK1G2* did not alter ERK signal in ER^+^ cells but slightly decreased in ER^-^ cells, another tumorigenesis-associated signal (Fig 5D–5H). ER^-^ MDA-MB-231 cells overexpressed with GFP-ERα mimicked the results of ER^+^ MCF-7 cells in activities of PI3K, AKT, mTOR, and rpS6 but not of ERK (Fig 5I–5K).

**Fig 6:**
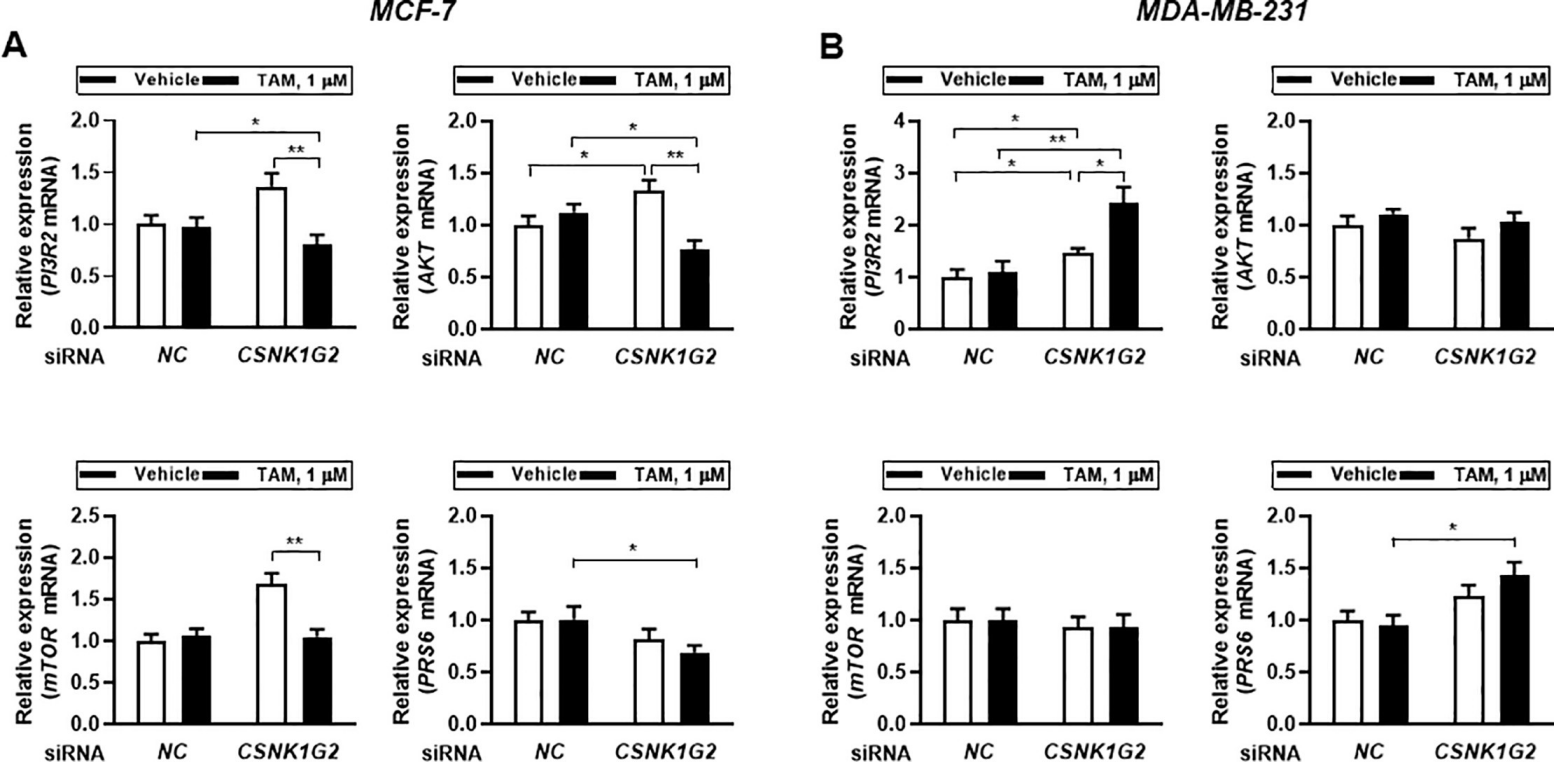
Effect of *CSNK1G2* on TAM-mediated PI3K/AKT/mTOR/S6K gene expression in human breast cancer cells. Transcriptional patterns of diverse genes, *PI3R2*, *AKT*, *mTOR*, and *PRS6*, were analyzed by RT-PCR in *CSNK1G2* silenced (A) ER^+^ and (B) ER^-^ breast cancer cells and compared with those in *NC* siRNA-transfected control. Knock-downed cells with *NC* or *CSNK1G2* siRNA were treated with vehicle or 1 μM of TAM for 24 h. All values were normalized to those in the *NC* siRNA-transfected vehicle group. *GAPDH* was used as a loading control. Data are presented as mean (SD); n = 5; *P < 0.05, **P < 0.01 *vs*. each represented counterpart.

The effect of the *CSNK1G2* knockdown on the expression of the genes involved in the aforementioned signaling was consistent with the results obtained from the western blot analysis. Specifically, in ER^+^ cells, *CSNK1G2* silencing suppressed TAM-mediated activated expression in *PI3R2*, *AKT*, and *PRS6* (Fig 6A), whereas in ER^-^ cells, *CSNK1G2* targeting enhanced the expression of *PI3R2* and *PRS6* following TAM treatment (Fig 6B). During the experiments, transfection efficiency of *CSNK1G2* was confirmed by real-time PCR. At this time, gene expression of other subtypes of *CSNK1G* such as *CSNK1G1* and *CSNK1G3* were also determined (S3 Fig). *CSNK1G2* silencing did not alter the expression of both *CSNK1G1* and *CSNK1G3* in MCF-7 cells but significantly enhanced *CSNK1G1* and *CSNK1G3* expression in ER^-^ cells. From the results, it is possible that the lack of TAM-sensitizing effects in ER^-^ cells might be partly due to the compensation action of enhanced *CSNK1G1* and *CSNK1G3* expression.

**Fig 7:**
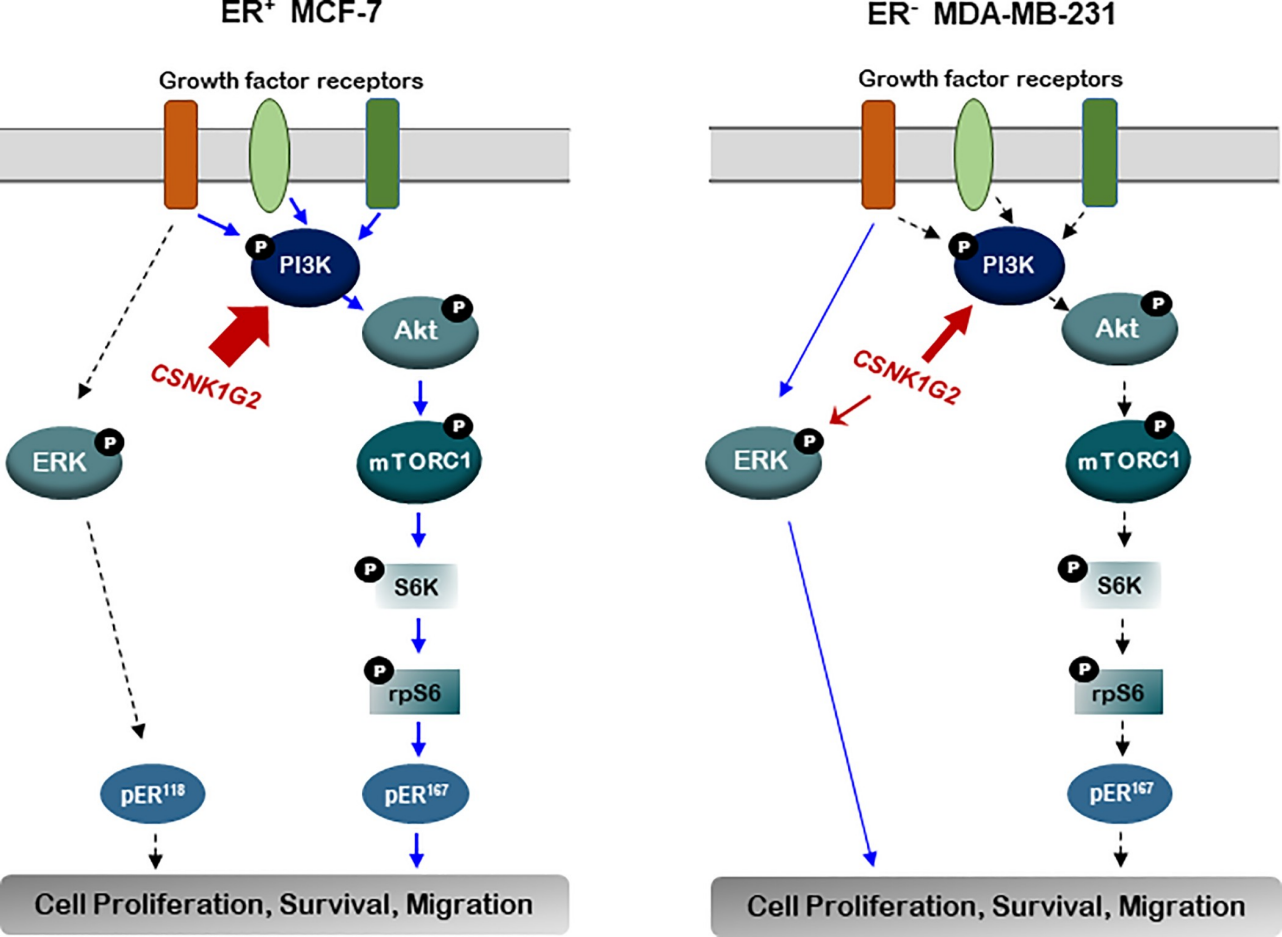
Schematic illustration of the *CSNK1G2*-regulated PI3K/AKT/mTOR/S6K signaling pathways in TAM treated human breast cancer cells. *CSNK1G2* differently influences PI3K/AKT and mTOR activity as well as S6K in ER^+^ and ER^-^ breast cancer cells. Red arrows represent the point of nodes altered by the *CSNK1G2*, and the blue arrows indicate the intracellular signal cascades activated by *CSNK1G2*. The thickness of the red arrow indicates the signal intensity. Abbreviations: **PI3K**, phosphatidylinositol 3-kinase; **mTOR**, mammalian target of rapamycin; **AKT,** protein kinase B; **rpS6**, ribosomal protein S6; **p-ER**^**118**^, phosphorylated estrogen receptor α at Ser^118^; **p-ER**^**167**^, phosphorylated estrogen receptor α at Ser^167^; **ERK**, extracellular signal-regulated kinase; and ***CSNK1G2***, *casein kinase 1 gamma 2*.

Taken together, in ER^+^ MCF-7 cells, *CSNK1G2* might modulate cytotoxicity via reinforced TAM-induced reduction in PI3K/AKT/mTOR/S6K, but not in ERK signaling cascades (Fig 7, left diagram). However, in ER^-^ MDA-MB-231, *CSNK1G2* only altered PI3K and ERK signals (Fig 7, right diagram). These results indicate that *CSNK1G2* may differently modulate breast cancer cells in a cell type-dependent manner.

### Casein kinase 1 inhibitor partly mimics *CSNK1G2* knockdown-mediated PI3K/AKT/mTOR/S6K signaling

**Fig 8:**
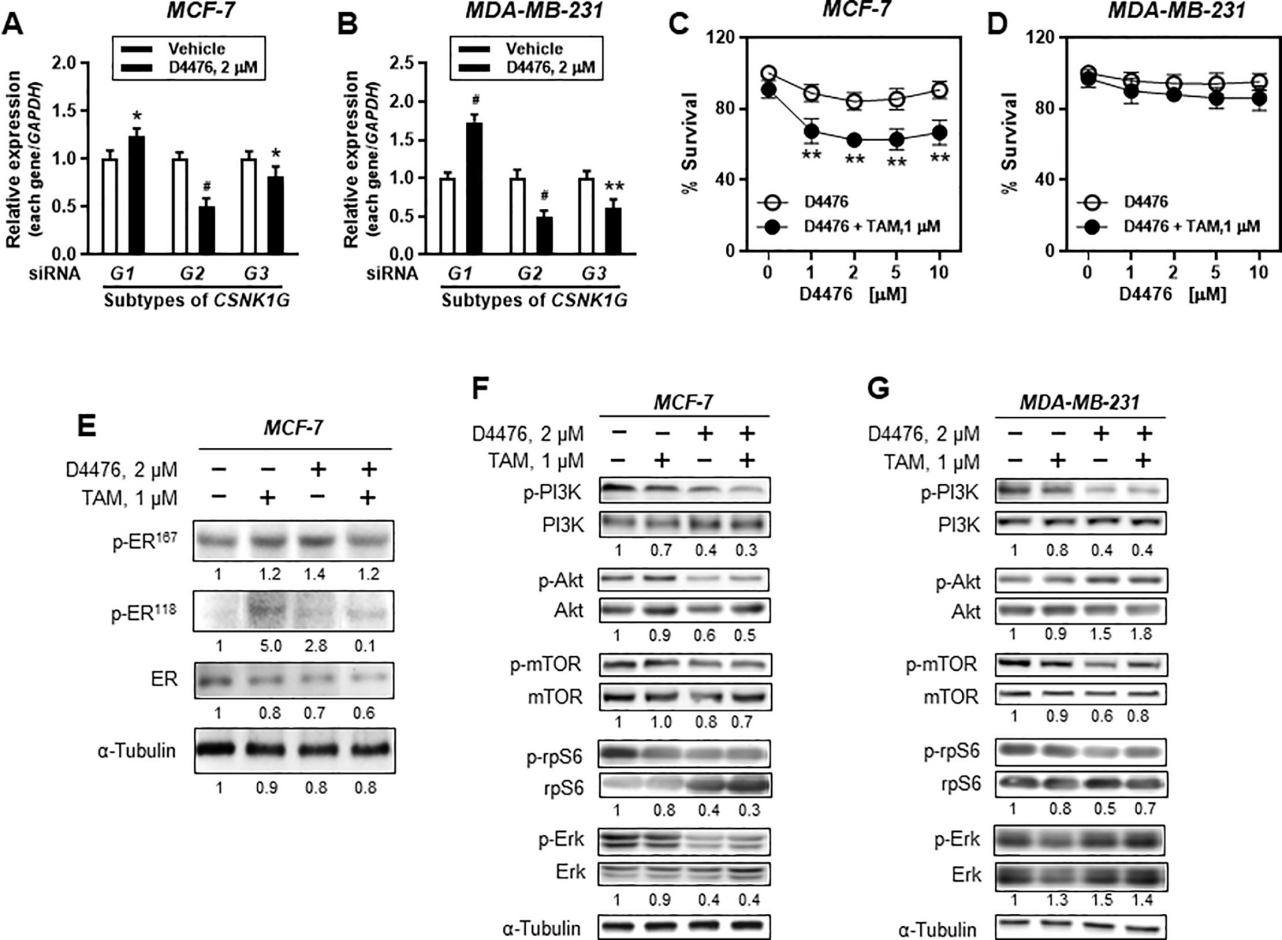
Casein kinase 1 inhibitor partly mimics *CSNK1G2* effect on PI3K/AKT/mTOR/S6K and ERK signaling in breast cancer cells. (A-B) Bars present relative mRNA expression of CSNK1G isotypes. After treatment with 2 μM D4476 for 24 h, (A) MCF-7 and (B) MDA-MB-231 silenced with *CSNK1G2* siRNA were analyzed for the mRNA expression of CSNK1G isotype genes such as *CSNK1G1*, *CSNK1G2*, and *CSNK1G3*. (C-D) Bars denote relative percentage of % survival of (C) ER^+^ or (D) ER^-^ breast cancer cells treated with D4476 alone or in combination with TAM for 24 h. MCF-7 and MDA-MB-231 cells were treated with 1 to 10 μM of D4476. For combination treatment, 1 μM TAM was also used. Cellular toxicity was measured using the MTT assay (mean (SD), n = 5; **P < 0.01 *vs*. each represented counterpart). (E-G) Western blotting analysis from the breast cells treated with vehicle or 2 μM D4476 together with 1 μM TAM for 24 h were performed. Immunoblots for (E) ERα and phospho-ERα (at Ser^118^ or Ser^167^) in MCF-7 cells and for PI3K/AKT/mTOR/S6K signaling-associated proteins in (F) MCF-7 and (G) MDA-MB-231 cells. *NC* siRNA or *CSNK1G2* siRNA-transfected cells were treated with 1 μM of TAM together with vehicle or with 2 μM of D4476 for 24 h. Protein expression levels based on individual bands of phosphor were compared to expression levels of α-tubulin and/or pan (total) protein. Each value under the blots indicates relative protein expression levels determined by densitometric analysis (mean ± SEM, n = 4).

Casein kinase 1 has many different subfamilies. In this study, we targeted the *CSNK1G2* subfamily. The subfamilies share common characteristics within cells, but different functions are also found among the subfamilies [15]. Therefore, we examined whether the effects that we found in TAM-treated breast cancer cells were unique to *CSNK1G2*. Breast cancer cells were exposed to 2 μM D4476, a CK 1 inhibitor, instead of *CSNK1G2* siRNA transfection. Before examining the cellular signals, we investigated which subtype was affected by D4476. Unexpectedly, in ER^+^ MCF-7 cells, D4476 significantly increased *CSNK1G1* expression but decreased both *CSNK1G2* (^#^*P*<0.001 *vs* vehicle) and *CSNK1G3* (*P<0.01 *vs* vehicle) with different degrees of significance (Fig 8A). Similar results were produced in ER^-^ MDA-MB-231 (Fig 8B). Additionally, when D4476-induced cytotoxicity was compared between ER^+^ MCF-7 and ER^-^ MDA-MB-231, cellular viability was significantly lower upon combined treatment of D4476 together with 1 μM TAM in ER^+^ cells than upon single treatment with 1 μM TAM (Fig 8C). However, ER^-^ cells did not show any significant cytotoxicity following combined treatment (Fig 8D).

Furthermore, when we examined the expression of cellular signal molecules, D4476 reproduced many of the effects of *CSNK1G2* on the PI3K/AKT/mTOR signaling system in breast cancer cells. However, unlike *CSNK1G2* knockdown, D4476 also altered ER phosphorylation at Ser^118^ but not at Ser^167^ and ERK activity in ER^+^ cells (Fig 8E and 8F). Different from MCF-7 cells, in the ER^-^ cells, D4476 slightly reduced PI3K activity, while increasing AKT and ERK activity (Fig 8G).

**Fig 9:**
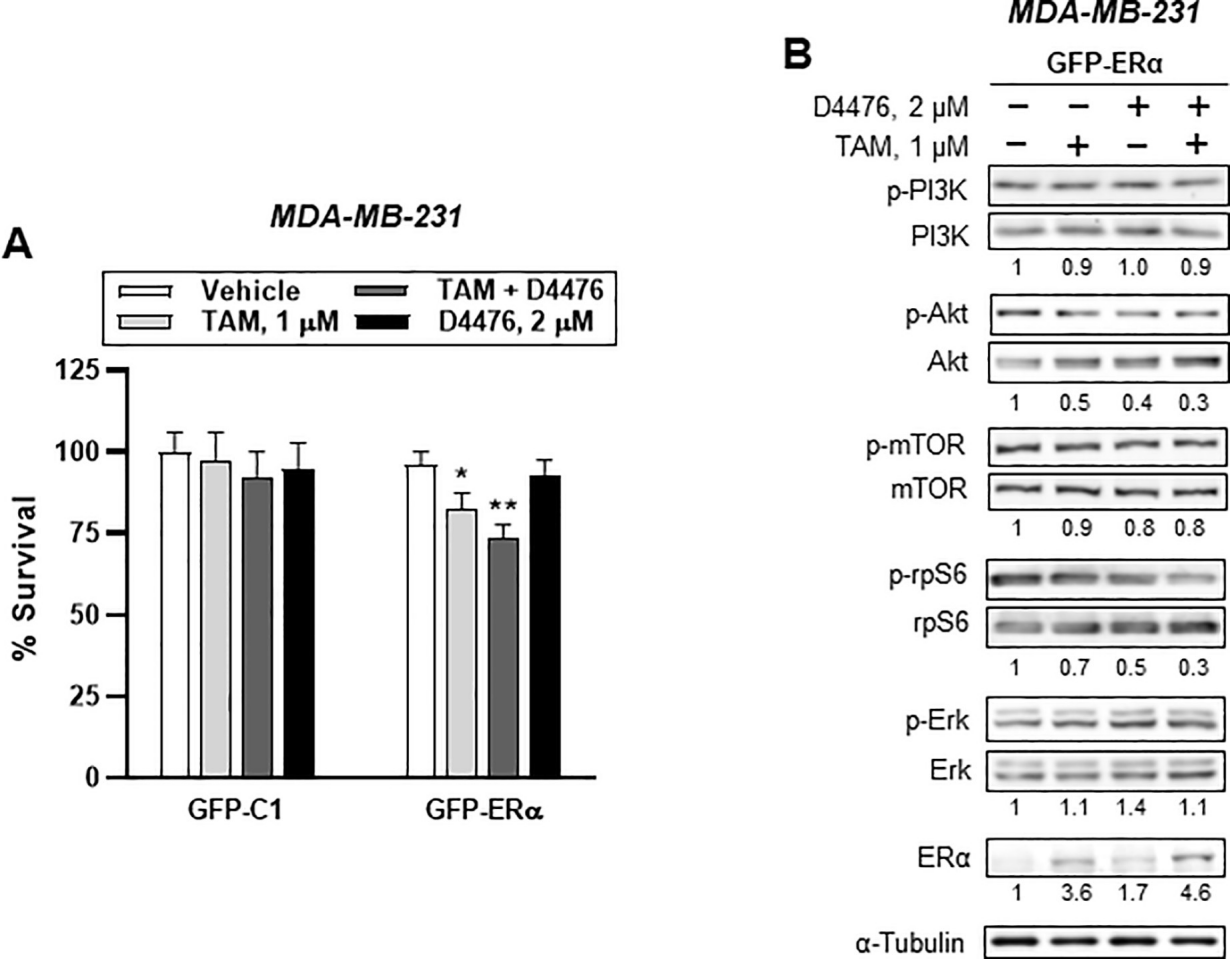
GFP-ERα overexpression partly mimics PI3K/AKT/mTOR/S6K and ERK signaling in ER^-^ breast cancer cells. (A) Cytotoxicity assay of either GFP-C1 or GFP-ERα-transfected ER^-^ breast cancer cells. Bar denote relative percentage of % survival of transfected MDA-MB-231 cells after treatment with vehicle, 1 μM TAM, 2 μM D4476, and 1 μM TAM plus 2 μM D4476 for 24h. Cellular toxicity was measured using the MTT assay (mean (SD), n = 5; *P < 0.05, **P < 0.01 *vs*. each represented counterpart). (B) Western blotting analysis from GFP-ERα-transfected ER^-^ breast cells treated with vehicle or 2 μM D4476 together with 1 μM TAM for 24 h were performed. Immunoblots for PI3K/AKT/mTOR/S6K signaling-associated proteins in ER^-^ MDA-MB-231 cells. Protein expression levels based on individual bands of phosphor were compared to expression levels of α-tubulin and/or pan (total) protein. Each value under the blots indicates relative protein expression levels determined by densitometric analysis (mean ± SEM, n = 4).

Interestingly, when ER^-^ MDA-MB-231 cells were overexpressed with GFP-ERα, MDA-MB-231 cells co-treated with D4476 and TAM showed similar pattern of TAM toxicity with the case of MCF-7 cells (Fig 9A). At the same time, activities of cellular signals such as PI3K, AKT, and rpS6 in GFP-ERα-transfected MDA-MB-231 were also similar with the MCF-7 response (Fig 9B). The findings indicated that the window of the targeting molecules by *CSNK1G2* is presumably narrower than that by D4476; thus, *CSNK1G2* can aim at a specific node of signal molecules. Moreover, ER existence partly contributes to *CSNK1G2* effects.

## Discussion

Target screening using a heterozygous gene deletion mutant library of *S*. *pombe* in this study reveals that nonessential genes (*cki3*, *rad24*, *sks2*) presently induce more sensitized cell growth than essential genes (*cdc2* and *cct6*). In the fission yeast genome, essential and nonessential genes are distributed evenly. Essential genes are significantly enriched for core cellular processes such as macromolecular (DNA, RNA, protein, and lipid) metabolism [20,25]. In contrast, nonessential genes noticeably enriched for regulatory functions including control of gene expression, cell communication, and conditional or life-cycle specific processes such as stress response, transmembrane transport, meiosis, and sexual reproduction [26]. Therefore, nonessential genes like *cki3* would be better drug targets for drug delivery because most genes indispensable for cellular survival should be protected for the viability of normal cells.

Breast cancer cells have heterogeneous characteristics. Therefore, ascertaining of the types of steroid receptors as well as assessment of multigene profiles that might influence a patient’s response to specific therapy should be first considered. ER signaling develops in various ways. ER activity in cells is largely due to the direct binding of dimeric ER to DNA sequences, EREs [27], which can accelerate transcriptional activity and mediate the expressions of various genes associated with protein synthesis, cell proliferation, and survival [28]. In addition, ER can indirectly associate with promoters via protein-protein interactions with other DNA-binding transcription factors. These processes are collectively termed genomic actions of estrogen [28]. Alternately, for non-genomic actions, ER associated with diverse receptors on plasma membrane such as G-protein coupled receptor, insulin growth factor receptor, and epidermal growth factor receptor can activate subsequent intracellular secondary messengers such as cAMP and other signal molecules, and initiate cellular signal cascades, ultimately resulting in indirect changes in gene expression [28].

TAM targets ER and attenuates the transcriptional activity in an ERE-dependent or non-ERE-dependent manner, eventually disrupting signaling pathways involved in tumor growth and migration [27,29]. In line with the drug action point of TAM, our experiment for TAM sensitivity in different breast cancer cell lines proved that ER^+^ cells responded sensitively to TAM, but ER^-^ cells showed poor reactivity. TAM can have antagonistic and agonistic effects against ER depending on the intracellular contents of the cells, thus it is important to discover new breast cancer targets that can reduce the adverse effects of TAM, while maintaining cytotoxicity to tumor cells [27]. In this study, the effect of *CSNK1G2* as a supplement of TAM was confirmed. The ERE activity as assessed by the reporter assay and analysis of the several genes such as *CTSD* and *GREB1* in MCF-7 cells raised the possibility that *CSNK1G2* partially contributes to the transcriptional activity of the ER-responsive genes through ERE regulation.

It has also been documented that *CTSD* and *GREB1* were increased in breast cancer patients [30,31]. In particular, CTSD expression is used as a prognostic marker, since the patients with high CTSD level resulted in poor overall survival and disease-free survival [30,31]. CTSD is active in low pH environments, stimulating tumor cells for pro-invasive and pro-metastatic capacities [32]. In hormone-responsive breast cancer cells, transcription of *CTSD* is also induced by estradiol but blocked by TAM [33]. Real-time PCR results in MCF-7 cells showed that the slightly upregulated expression of estrogen-responsive genes following treatment with low concentration of TAM was almost completely returned to the normal level following target gene knockdown. However, it is not yet clear how *CSNK1G2* is involved in a series of processes such as ERα activation and subsequent activation of ER-responsive genes.

CSNK1G is ubiquitously expressed in a wide range of eukaryotes ranging from yeasts to humans [34]. In contrast to yeast CSNK1 isoforms, the functions of mammalian CSNK1 isoforms remain poorly characterized. CSNK1 can interact with a large number of substrates involved in multiple cellular functions, including regulation of membrane trafficking, vesicular transport, cytokinesis, ribosome biogenesis, DNA repair, signal transduction pathways, and circadian rhythm [13]. Many reports have described the regulation of CSNK1 isoforms, including MDM2 stability of p53 by CSNK1A, and control of Wnt/β-catenin and hedgehog signaling by CSNK1E and CSNK1D [35,36]. However, the roles of CSNK1G isoforms have yet to be extensively investigated and remain unclear. Only enhanced expression of CSNK1G3 in renal cell carcinoma has been reported [37]. Analysis of the actual datasets of the cancer genome atlas (TCGA), available at the cBioPortal for Cancer Genomics [38,39], revealed the mutation pattern and copy number alterations of CSNK1 subtypes in 24 different tumor types. The analysis focused only on CSNK1A1, CSNK1D, and CSNK1E, not on CSNK1G; CSNK1A1 alterations are dominant in clear cell renal cell carcinoma, and pancreas cancer, CSNK1D in liver and sarcoma, and CSNK1E in melanoma and liver.

As presented by the expression of *CD44*/*CD24* and tumor sphere formation in MCF-7 and MDA-MB-231, *CSNK1G2* also affected the expression of breast stem cell-markers only in ER^+^ breast cancer cells. The combination of CD44 and CD24 or aldehyde dehydrogenase 1 (ALDH1) is a representative cancer stem cell marker in breast cancer [40,41]. Breast stem cells potentially have the ability of replication and tumor formation, thus leading to metastasis from one organ to another [40,41]. Indeed, a xenograft study using nude mice and other *in vivo* experiments have reported that disseminated tumor cells in the bone marrow of breast cancer patients showed a high CD44^+^/CD24^-^ ratio [42] and the elevated expression of CD44^+^/CD24^-^ was closely associated with lung metastasis [41].

Phosphorylation of ERα is essential for its activation after stimulation with various ligands and nonsteroidal growth factors. Phosphorylation of ERα at Serine (Ser)^118^ in the NH_2_ terminus within the AF-1 region is mediated by the CDK7 [43] and Ras/MAPK pathways [44,45], which enable ligand-independent transactivation of ERα by interaction with the transcriptional coactivators, CBP and SRC-1. However, phosphorylation at Ser^167^ in AF-1 is associated with several protein kinases including casein kinase II, p90 RSK (p90 ribosomal S6 kinase), AKT, and p70 RSK, thereby enhancing DNA binding and transcriptional activity [46,47]. In our study, *CSNK1G2* silencing significantly suppressed E_2_-triggered phosphorylation of ERα at both Ser^118^ and Ser^167^, but it only reduced TAM-mediated ERα phosphorylation at Ser^167^. This is very important because phosphorylation of ERα at Ser^167^ is indicative of prolonged disease-free and overall survival in breast cancer patients [48]. Furthermore, when the activity was lost by *ERα* siRNA, *CSNK1G2* silencing-induced TAM sensitivity was completely blocked. However, it is not clear whether the effect of *CSNK1G2* on ERα activity occurs via direct interaction to ERα or via any connection with other substrates such as AKT, RSK, or S6K.

Representative signaling pathways known to be involved in tumor growth include PI3K/AKT/mTOR, and Ras/Raf/MAPK [49], which are associated with stimulation of proliferation and protection from apoptosis [50]. Hyperactivation of PI3K/AKT/mTOR pathway drives tumorigenesis of ER^+^ breast cancer [51,52] and resistance to endocrine therapy [53,54]. Therefore, new approaches with various inhibitors of the PI3K/AKT/mTOR pathway, such as everolimus and exemestane, augment the benefit of existing endocrine therapy by extending time to disease progression and preventing or overcoming resistance to endocrine treatment [55]; thus, many preclinical and clinical studies are currently focusing on this. AKT has been focused on as a therapeutic target for cancer as it works as a nodal point linking cell growth, apoptosis, ribosomal biogenesis, and cellular metabolism [56]. The AKT3 targeting strategy is specifically effective for ER-negative, androgen receptor (AR)-negative breast or prostate cancer cells [57]. Interestingly, a previous proteomic study demonstrated that breast cancer cell line-specific associations with specific signals exist. For example, luminal B subtype of human breast cancer hyperactivates PI3K signaling associated with lower ER levels, but PI3K blockade increases ER level. Therefore, dual targeting of the PI3K and ER signaling pathways may be useful in patients with ER^+^ tumors [58]. Conversely, basal-like breast cancer displays greater sensitivity to selective MEK inhibition than luminal or HER2^+^ subtypes [59]. Together with PI3K pathways, Raf/MEK/ERK pathway is highly conserved and transmits signals from the cell surface receptors to nuclear transcription factors. Of the three, ERK is specially focused on crosstalk with PI3K signals. ERK1/2 converges on mTORC1 via p90S6K, thus influencing cell proliferation, survival, motility, and angiogenesis [12].

Recently, treatment-predictive role and prognostic value of the S6K has been also reported. Aberrant expression and localization of S6K are characterized as a poor sign for ER^+^ patients who require TAM treatment [56]. In accordance with this, S6K1 has been shown to phosphorylate the ER at Ser^167^, inducing conformational changes in the receptor and making it less responsive to TAM [60]. We here observe that, in breast cancer cells, *CSNK1G2*-silenced cells lead to the suppression of not only PI3K/AKT/mTOR/S6K but also ERK. Although attenuation of ERK signal was only seen in ER^-^ breast cancer cells, it is interesting that *CSNK1G2* can target both PI3K/AKT/mTOR/S6K and ERK signal pathways depending on the characteristics of hormone receptor subtypes. *CSNK1G2* selectively manipulates the tumor progress, sometimes in a nuclear and genomic manner via ERE inhibition of specific genes associated with tumor growth, but sometimes by non-genomic manner via the AKT/mTOR/S6K pathway, followed by p-ER^167^ activity. However, these effects can occur simultaneously, thus leading to strong suppression.

Many chemotherapies that utilize selective ER modulators suppress cell growth-associated signals, but their effects do not last, owing to the activation of multiple compensatory mechanisms [61]. This leads to adverse effects of cancer therapy. Nonetheless, our results indicate that *CSNK1G2* induces complete reduction in most signals ranging from PI3K to S6K; even these inhibitory effects on cell growth were ER-dependent. In addition, we presently observed that S6 kinase signaling, the downstream of AKT-mTOR, was the most strongly reduced by the combined treatment with TAM and *CSNK1G2* silencing. Interestingly, however, many reports demonstrated that the continuous suppression of S6 kinase can activate upstream signals such as AKT and PI3K signaling, thereby hindering the inhibition of cancer progress [56]. This is because there are multiple crosstalk and interconnections present in the PI3K/AKT/mTOR/S6K and ERK signaling pathways; thus, suppression of the specific signals may lead to activation in negative feedback regulatory mechanisms. Consequently, this may provide unwanted activation of signaling molecules such as AKT and ERK, upon inhibition [12,62]. However, we observed that the reduction of S6 kinase by *CSNK1G2* in ER^+^ cells did not activate upstream signaling molecules such as PI3K, AKT, and mTOR, as well as ERK, indicating the possible role of CSNK1G2 in specific cellular signaling control without interruption of other signals. Moreover, the study using D4476 also proved that *CSNK1G2* does not follow all the effects of CK1, and instead displays its own effects via different targeting points from D4476.

Taken together, our results reveal a unique action mechanism of *CSNK1G2* on PI3K/AKT/mTOR/S6K and ERK signaling pathways in breast cancer cells and provide insight into how the target selectively controls and contributes to cell survival in different human breast cancer cell types. Furthermore, these results provide a rationale for collectively assessing cellular signals for improved prognosis in clinical trials of breast cancers treated with inhibitors of PI3K and MAPK pathways.

## Supporting information

S1 Fig Expression of CSNK1G2 in *CSNK1G2* siRNA-transfected ER^+^ and ER^-^ breast cancer cells. Immunoblots for *CSNK1G2* siRNA-transfected breast cancer cell lines. Gene knockdown efficiency in individual breast cancer cell lines was determined by western blot analysis (mean (SD); n = 5). Protein expression levels were compared to expression levels of α-tubulin; each value under the blots indicates relative protein expression levels determined by densitometric analysis.
(TIF)

S2 Fig Effect of *CSNK1G2* on PI3K/AKT/mTOR/S6K signaling in E_2_-treated ER^+^ and ER^-^ breast cancer cells. Immunoblots for PI3K/AKT/mTOR/S6K signaling-associated proteins in (A) MCF-7 and (B) MDA-MB-231 cells. Western blot analysis from the breast cells transfected with control siRNA (*NC*) or *CSNK1G2* siRNA (*CSNK1G2*) were performed after treatment with vehicle or 10 nM E_2_ for 24 h. Protein expression levels based on individual bands of phosphor were compared to expression levels of α-tubulin and/or pan (total) protein. Each value under the blots indicates relative protein expression levels determined by densitometric analysis. Data are expressed as mean (SD); n = 4.
(TIF)

S3 Fig mRNA expression of *CSNK1G* subtypes following *CSNK1G2* silencing in ER^+^ and ER^-^ breast cancer cells. Quantitative analysis of mRNA expression in *NC* siRNA- or *CSNK1G2* siRNA-transfected (A, B) MCF-7 cells and (C, D) MDA-MB-231. *CSNK1G1* (A, C) and *CSNK1G3* (B, D) expression levels were determined 24 h after 1 μM TAM treatment. *GAPDH* was used as a loading control. Data are expressed as mean (SD); n = 4; *P < 0.05, **P < 0.01, ^#^*P* < 0.001 *vs*. each represented counterpart.
(TIF)

S1 Table Real-Time PCR primer sequences. (DOCX)

S1 Raw images (TIF)

S2 Raw images (TIF)
