# Supplementary figures and images for: CSNK1G2 differently sensitizes tamoxifen-induced decrease in PI3K/AKT/mTOR/S6K and ERK signaling according to the estrogen receptor existence in breast cancer cells
Source: PLoS One. 2021 Apr 16;16(4):e0246264. doi: 10.1371/journal.pone.0246264 (PMC8051802; doi:10.1371/journal.pone.0246264)

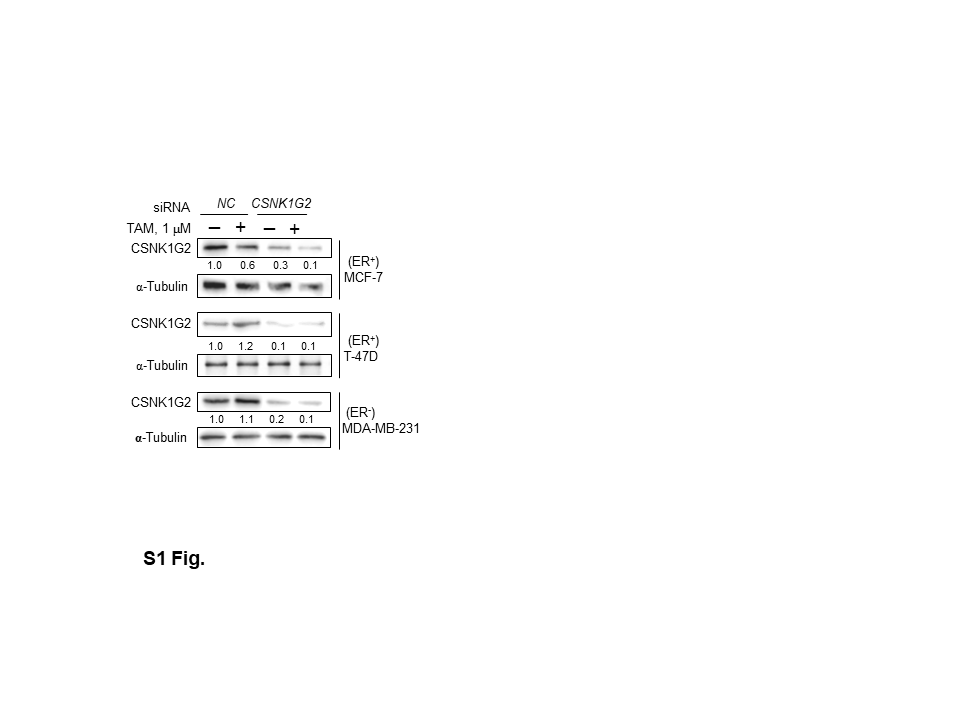

Supplement: S1 Fig — Immunoblots for CSNK1G2 siRNA-transfected breast cancer cell lines. Gene knockdown efficiency in individual breast cancer cell lines was determined by western blot analysis (mean (SD); n = 5). Protein expression levels were compared to expression levels of α-tubulin; each value under the blots indicates relative protein expression levels determined by densitometric analysis. (TIF) [file pone.0246264.s001.tif]

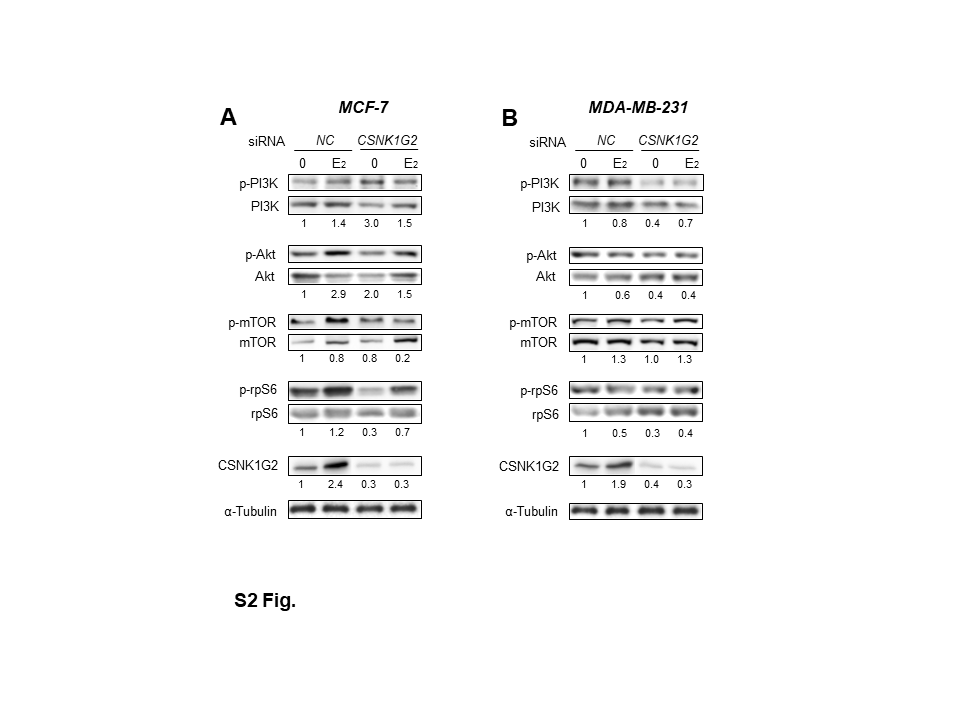

Supplement: S2 Fig — Immunoblots for PI3K/AKT/mTOR/S6K signaling-associated proteins in (A) MCF-7 and (B) MDA-MB-231 cells. Western blot analysis from the breast cells transfected with control siRNA (NC) or CSNK1G2 siRNA (CSNK1G2) were performed after treatment with vehicle or 10 nM E2 for 24 h. Protein expression levels based on individual bands of phosphor were compared to expression levels of α-tubulin and/or pan (total) protein. Each value under the blots indicates relative protein expression levels determined by densitometric analysis. Data are expressed as mean (SD); n = 4. (TIF) [file pone.0246264.s002.tif]

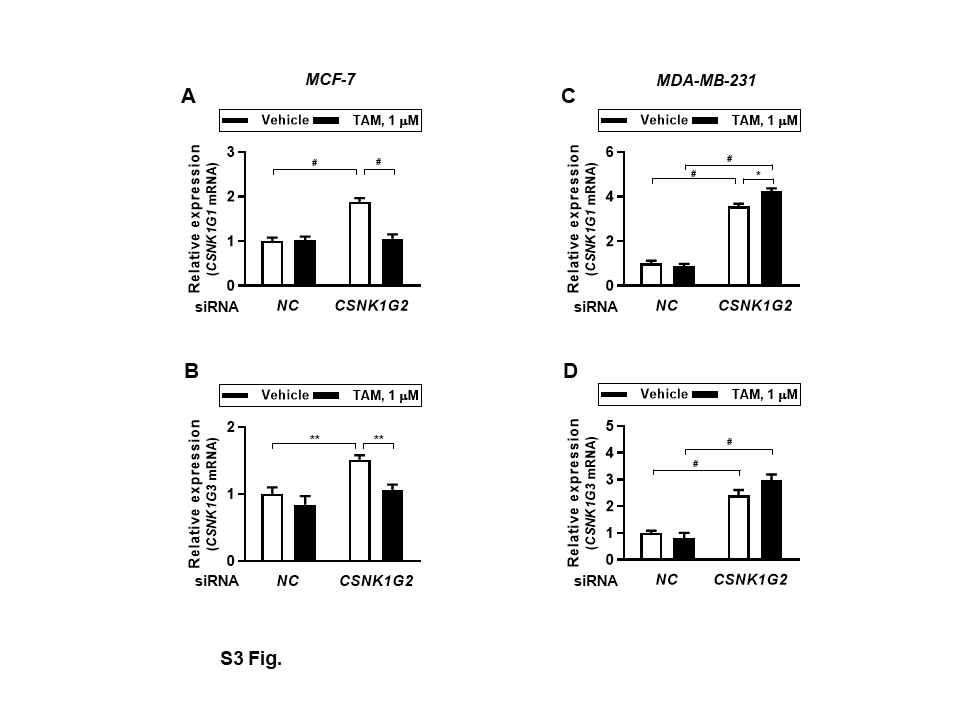

Supplement: S3 Fig — Quantitative analysis of mRNA expression in NC siRNA- or CSNK1G2 siRNA-transfected (A, B) MCF-7 cells and (C, D) MDA-MB-231. CSNK1G1 (A, C) and CSNK1G3 (B, D) expression levels were determined 24 h after 1 μM TAM treatment. GAPDH was used as a loading control. Data are expressed as mean (SD); n = 4; *P < 0.05, **P < 0.01, #P < 0.001 vs. each represented counterpart. (TIF) [file pone.0246264.s003.tif]

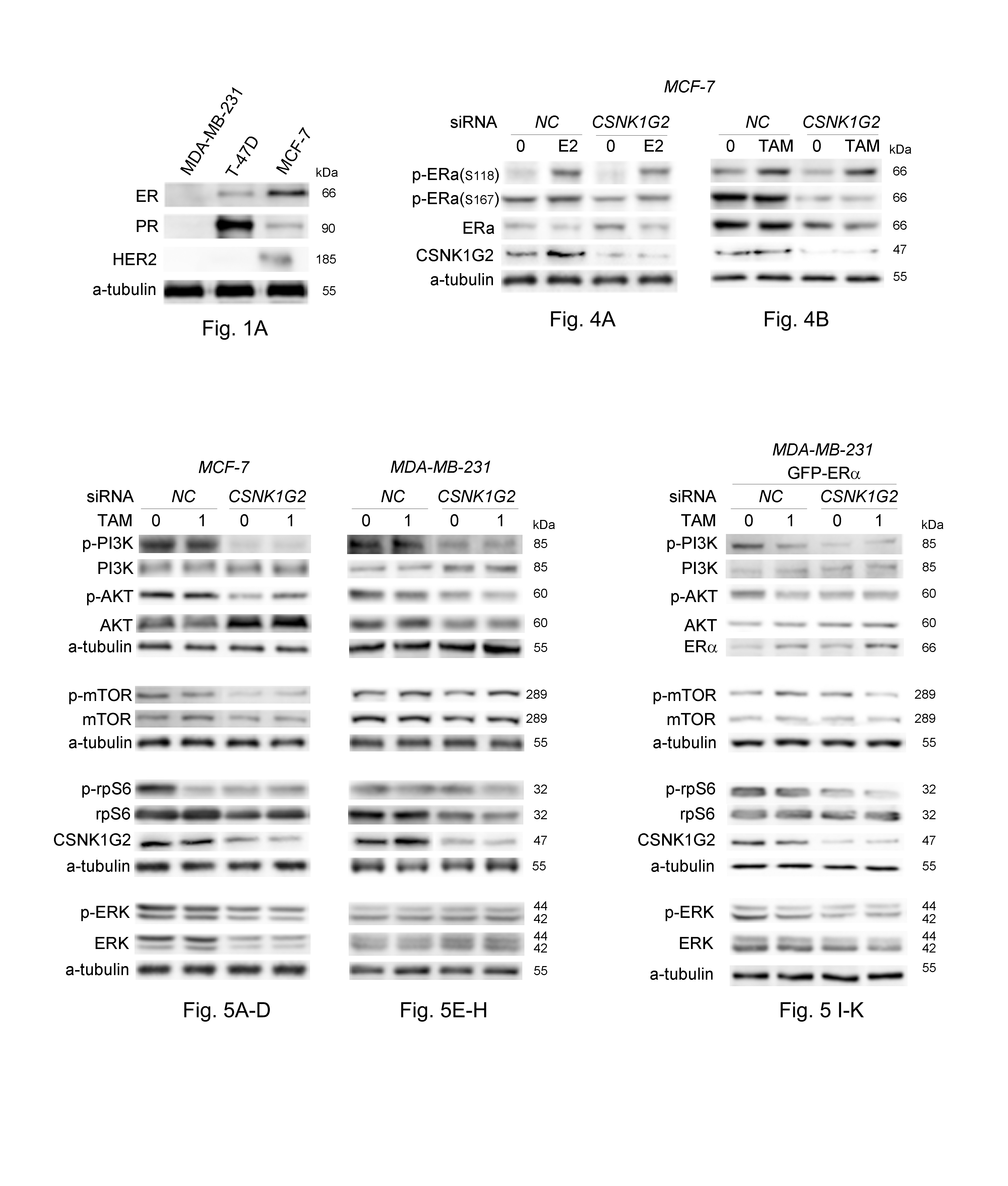

Supplement: S1 Raw images — (TIF) [file pone.0246264.s005.tif]

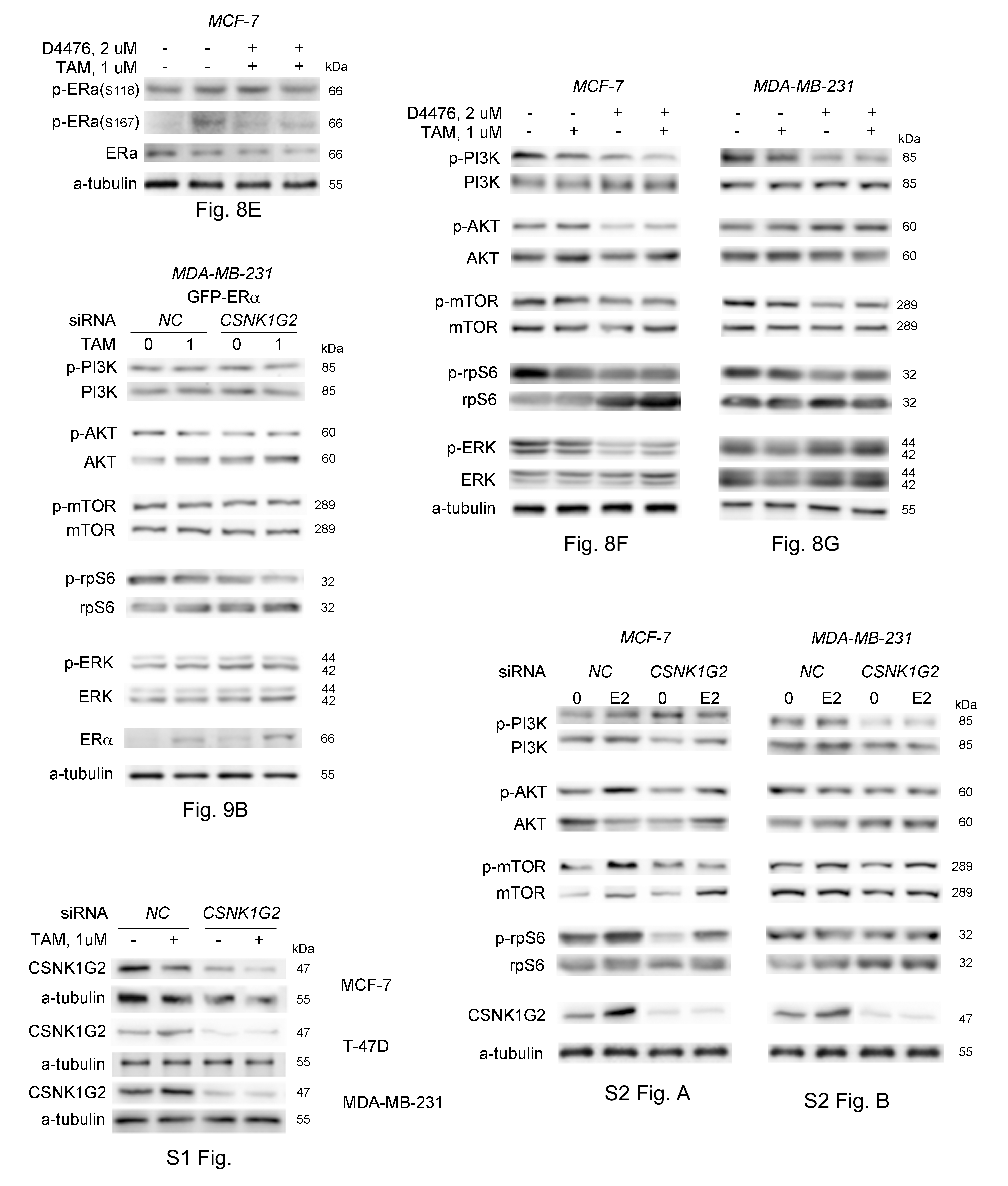

Supplement: S2 Raw images — (TIF) [file pone.0246264.s006.tif]
