# Supplementary material for: CSNK1G2 differently sensitizes tamoxifen-induced decrease in PI3K/AKT/mTOR/S6K and ERK signaling according to the estrogen receptor existence in breast cancer cells
Source: PLoS One. 2021 Apr 16;16(4):e0246264. doi: 10.1371/journal.pone.0246264 (PMC8051802; doi:10.1371/journal.pone.0246264)
Supplement: S1 Table — (DOCX) [file pone.0246264.s004.docx]

**S1 Table. Real-Time PCR primer sequences**

| Genes | Forward primer sequence (5’-3’) | Reverse primer sequence (5’-3’) |
| --- | --- | --- |
| *AKT* | GTCATCGAACGCACCTTCCAT | AGCTTCAGGTACTCAAACTCGT |
| *CD24* | CTCCTACCCACGCAGATTTATTC | AGAGTGAGACCACGAAGAGAC |
| *CD44* | CTGCCGCTTTGCAGGTGTA | CATTGTGGGCAAGGTGCTATT |
| *CSNK1G1* | ATGGACCATCCTAGTAGGGAAAA | CACATCCTATCTTCTTGCCAACC |
| *CSNK1G2* | CAAATTGGAGCCGATCAAGT | GGTGTGCACATACTCCATGC |
| *CSNK1G3* | TGGAGATACAAAACGGGCTACA | GCAACTGTTTACCAATCCAGTCA |
| *CTSD* | TGCTCAAGAACTACATGGACGC | CGAAGACGACTGTGAAGCACT |
| *GREB1* | ATGGGAAATTCTTACGCTGGAC | CACTCGGCTACCACCTTCT |
| *mTOR* | GAGGTGAATTGAGGTCCCTAAGA | ATGCTTGGAACCGGACCTG |
| *PIK3R2* | TCACCTTCTGCTCCGTTGTG | GGAGGTCCGTGTGTACTCTTC |
| *PS2* | AGTGTGTGATGAGCGGACG | ACTGGGCAGTGTTCTCTCCAT |
| *RPS6* | AGGGTTATGTGGTCCGAATCA | TTGGTCTGTAACAGGAATGCC |
